# Effects of nature-adapted lighting solutions (“Virtual Sky”) on subjective and objective correlates of sleepiness, well-being, visual and cognitive performance at the workplace

**DOI:** 10.1371/journal.pone.0288690

**Published:** 2023-08-03

**Authors:** Isabel Schöllhorn, Gunnar Deuring, Oliver Stefani, Michael A. Strumberger, Timm Rosburg, Patrick Lemoine, Achim Pross, Benjamin Wingert, Ralph Mager, Christian Cajochen

**Affiliations:** 1 Centre for Chronobiology, Psychiatric Hospital of the University of Basel, Basel, Switzerland; 2 Research Platform Molecular and Cognitive Neurosciences (MCN), University of Basel, Basel, Switzerland; 3 Forensic Department, Basel University, University Psychiatric Clinics, Basel, Switzerland; 4 Department of Clinical Research, Division of Clinical Epidemiology, EbIM Research & Education, University of Basel Hospital, Basel, Switzerland; 5 Fraunhofer-Institute for Industrial Engineering, Stuttgart, Germany; Julius-Maximilians-Universität Würzburg, GERMANY

## Abstract

Exposure to natural daylight benefits human well-being, alertness, circadian rhythms and sleep. Many workplaces have limited or no access to daylight. Thus, we implemented a light-panel (“Virtual Sky“), which reproduced nature-adapted light scenarios. In a laboratory office environment, three lighting scenarios were presented during the day: two lighting conditions with nature-adapted spectral light distributions, one with static and one with dynamic clouds, and a standard office lighting condition. We compared the impact of the three lighting scenarios on subjective and objective measures of alertness, cognitive performance, wellbeing, visual comfort, contrast sensitivity, and cortisol levels in 18 healthy young male volunteers in a within-participant cross-over study design. We found no evidence that an 8-h lighting scenario with static or dynamic clouds during the waking day (9am-5pm) was associated with any significant effect on objective and/or subjective alertness, cognitive performance and morning cortisol concentrations compared to standard workplace lighting. However, the dynamic light scenario was accompanied with lower levels of perceived tensionafter completing cognitive tasks and less effort to concentrate compared to the static lighting scenarios. Our findings suggest that apart from smaller effects on tension and concentration effort, nature-adapted lighting conditions did not improve daytime alertness and cognitive performance in healthy well-rested young participants, as compared to standard office lighting.

## Introduction

Nowadays, in modern societies, people spend a large part of their time inside buildings with limited access to daylight. According to Hubert et al., many of us spent more than 50% of their time awake in lighting conditions below 100 lux [1], which corresponds to just 0.1 percent of daylight. Light during the day is essential for proper synchronization of the body`s circadian clock with the environmental light-dark cycle [2]. Thus, the lack of natural daylight increases the risk of circadian misalignments, winter depression (Seasonal Affective Disorder) and sleep disturbances [3, 4].

These non-image forming (NIF) effects of light on the human circadian timing system and sleep are mediated via the intrinsically photosensitive retinal ganglion cells (ipRGCs) that use the photopigment melanopsin to absorb light [5]. IpRGCs are directly connected via retinohypothalamic pathways to the suprachiasmatic nuclei in the anterior hypothalamus [5, 6], the central pacemaker for circadian rhythms. Furthermore, ipRGCs are mostly sensitive to visible short wavelength radiation (~490 nm after pre-receptoral filtering) [7], which is close to the peak of the daylight spectrum (510 nm). Besides photoentrainment through ipRGCs, the central pacemaker located in the suprachiasmatic nuclei also receives transient input from cones at high frequency modulations in irradiance, as it can occur in nature, for example if clouds obscure the sun [8].

The effects of daylight on circadian entrainment and on mood are well documented [9]. There is some evidence that natural lighting that mimics the characteristics of daylight (i.e. brightness, spectrum, dynamics) has effects on mood and circadian entrainment, similar to those of natural daylight, and, possibly secondary to these effects, positive effects on cognitive performance: Bright light during the day (> 1000 lx) can increase subjective and objective correlates of alertness and performance [10–15]. Furthermore, there is evidence that blue-enriched white light with high colour temperatures (17000 K, comparable to natural skylight) can positively affect mood, increase performance and alertness levels compared to standard office white light (4000 K) [15]. Nevertheless, daytime might modulate such effects: light induced alerting effects were reported to be less pronounced, more inconsistent, and less conclusive during the day than in the evening and at night [16].

The characteristics of standard artificial office lighting and daylight differ in many ways, such as brightness, short wavelength components, the size of light sources, and the dynamic fluctuations across the day. During dawn and dusk, the amount of light, the spectral composition, and sun’s position change systematically. Furthermore, acute changes in the natural light environment can occur, for example when a cloud covers the sun [17]. A constant change of brightness and spectral composition in the morning while waking up may increase cognitive performance, mood and well-being compared to constant dim light [18]. Studies investigating dynamic lighting in offices have shown that slow changes in direct and indirect light components [19], illuminance and spectrum [20, 21] can increase well-being and motivation [19]. However, the evidence is limited and inconclusive. Some studies could not find positive effects on well-being and cognitive performance when correlated colour temperature and/or illuminance levels were changed [22, 23]. Previous research using photos, videos or VR to present natural scenes such as forests or water could show positive effects on respiration patterns and stress reduction [24, 25]. Stefani et al. examined a cloud simulation with and without dynamics in an office environment. Under the dynamic light condition, subjective well-being was better, and subjective fatigue was reduced in the afternoon. Furthermore, the study participants preferred the dynamic to static light during creative work [20].

It needs to be better understood whether and to what extent acute nature-adapted lighting at work places affect work performance, well-being, and circadian physiology. Previous research revealed an acute effect of light exposure on cortisol levels (e.g. [26–28]) and suggest that morning bright light elevates morning cortisol levels and increases alertness [26]. However, there is limited evidence about the influence of different light characteristics (e.g. illuminance, spectrum) or light patterns (dynamic vs. static) on cortisol.

Current lighting standards for workplaces that focus on visual performance (e.g. DIN EN 12464–1) [29] do not consider NIF effects of light and mainly focus on characteristics like illuminance, glare, or colour rendering. (for detailed discussion, see [30]) Therefore, specified illuminance (e.g. horizontal illuminance of 500 lx) and Correlated Colour Temperature (warm to neutral white: 3300 to 4000 K) are often not sufficient to reach the recently recommended 250 lx melanopic equivalent daylight illuminace (mEDI) during daytime [31]. These recommendations are based on a detailed sensitivity analysis of the human circadian, neuroendocrine, and alerting responses to ocular light.

Here, we aimed to assess whether optimizing the spectrum by increasing Correlated Color Temperature positively affects objective and subjective measures of alertness, well-being, cognitive and visual performance, and the stress hormone cortisol. Moreover, we investigated the effects of cloud imitating light dynamics on these NIF effects. In a crossover design, study participants were exposed to three different light conditions: one static (SC = static clouds) and one dynamic (DC = dynamic clouds) nature-adapted light condition, as well as a standard office lighting (SL) as a control condition. The primary aim of this study was to test whether nature-adapted lighting scenarios (“Virtual Sky”) outperform standard lighting conditions in their impact on alertness, well-being, cognitive and visual performance, and cortisol correlates of stress levels at the workplace. One of our previous studies (Cajochen et al., 2011) inspired our selection of tasks. We tested several domains that might be relevant to different types of office work. We considered such broad testing important to rule out that positive lighting effects in one domain might have negative effects elsewhere, such as improved attention but higher tension, even if such negative effects were not expected.

Specific Hypotheses:

1. Compared to a standard office lighting condition (SL), nature-adapted spectrum (SC and DC) lead to an increase in subjective and objective alertness. Dynamic changes of light levels enhance alerting effects (SL<SC<DC).
2. Compared to a standard office lighting condition (SL), nature-adapted spectrum (SC) and dynamic changes (DC) attenuate the decrease of cognitive performance during daytime, as indexed by response times, sustained attention, response control, and declarative memory (SL<SC<DC).
3. Nature-adapted spectrum and light dynamics improve levels of well-being, mood, visual comfort, subjective effort and increase salivary cortisol levels in the morning.
4. Light dynamics might reduce contrast sensitivity.

## Method

### Participants

After giving written informed consent, 18 healthy male volunteers (age range: 20–33 years, mean age: 23.2 ± 3.7 years) successfully completed the study. All volunteers received monetary compensation for their participation in the study. The study was carried out in the Centre for Chronobiology in Basel (Switzerland), between September 2016 and May 2017. The study protocol, screening questionnaires and consent form were approved by the Ethics Committee Northwest/Central Switzerland (EKNZ, PB_2016–02023) and conformed to the Declaration of Helsinki. All study participants had a good sleep quality as assessed with the Pittsburgh Sleep Quality Index [32] (PSQI score ≤ 5) and were not extreme chronotypes as assessed via the Munich Chronotype Questionnaire in particular the mid-sleep on free days (MSF) corrected for sleep debt on work days (‘MSF_sc_’, 2 < MSF_sc_ < 7) [33]. They underwent an ophthalmic examination by a certified optometrist to exclude volunteers with visual impairments. This included, testing visual acuity and colour vision (‘Ishihara Test’) [34]. We included participants whose refractive error was corrected by glasses or contact lenses. Exclusion criteria were smoking, medication or drug consumption, shift work within the last three months, and transmeridian flights up to one month prior to the study.

Participants were required to maintain a regular sleep-wake schedule (± 30 minutes) for one week before the first day of experiment and during the 1-week wash-out periods between the three experimental days. To ensure compliance to this protocol, actimetry recordings of motor activity of the non-dominant arm were assessed. Additionally, participants reported their sleep and wake episodes using a sleep-wake diary (like a questionnaire). Immediately after awakening, seven items had to be completed (bedtime, lights-off, estimated sleep onset latency, number of waking up during night, number of getting up during night, wake-up time/lights-on, and getup time in the morning). Moreover, they had to rate the sleep quality, restorative sleep, and sleepiness before lights-off on a Likert scale (1 = “very poor” to 8 = “very good”).

### Study design

**Fig 1:**
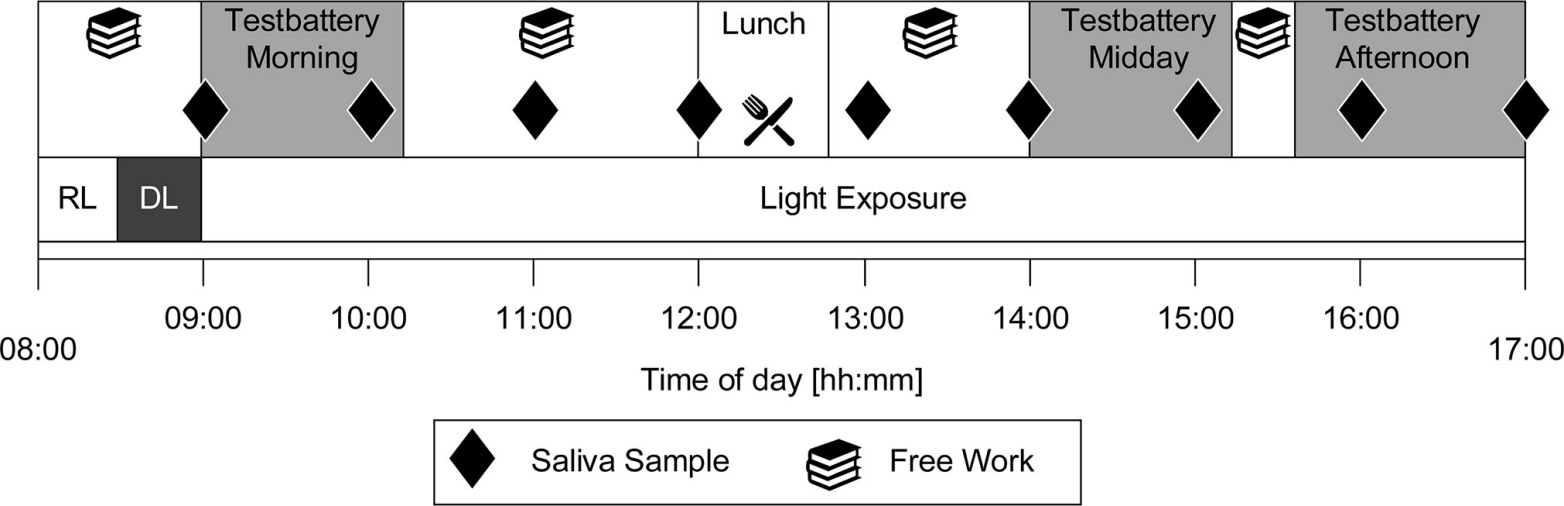
Schematic illustration of the study protocol of an experimental day, during which each participant was exposed to one of the three light conditions. Before the light exposure, electroencephalographic (EEG) electrodes were attached in room light (RL). Subsequently, participants remained in dim light (DL) for 30 minutes. Saliva samples were collected in hourly intervals. Participants completed three test batteries during each experimental day. Between test assessments, participants were allowed to do office work of their own choice with printed media.

The study consisted of three experimental days, separated by a 1-week intervening period and was conducted in a controlled lab environment (Fig 1). Participants were exposed to one of the three light conditions on each experimental day in a balanced order. The experimental protocol started at 8:30 a.m. with 30 minutes of light adaptation, after which the participants were exposed to one of the three lighting scenarios over a period of 8 hours each. At three different time points (morning, midday, afternoon) of each experimental day, a test-battery assessed cognitive performance, subjective variables, and contrast sensitivity. Additionally, saliva samples were taken every 60 minutes.

During their stay in the lab, participants were predominantly in a sitting position in constant environmental conditions (i.e. room temperature ~ 21° C) with no external time cues, including clocks, radios, visitors, and sunlight. Lunch was provided at midday 12 PM. During the free working blocks, participants were allowed to read their own books, magazines, and other documents. They were also allowed to stand up and relax with open eyes for up to 10 minutes. It was forbidden to use electronic devices (e.g. laptops or mobile phones), to sleep, or to listen music. After each block of free working, subjects received a test sheet to evaluate how stimulating and pleasant the reading was.

### Virtual Sky

**Fig 2:**
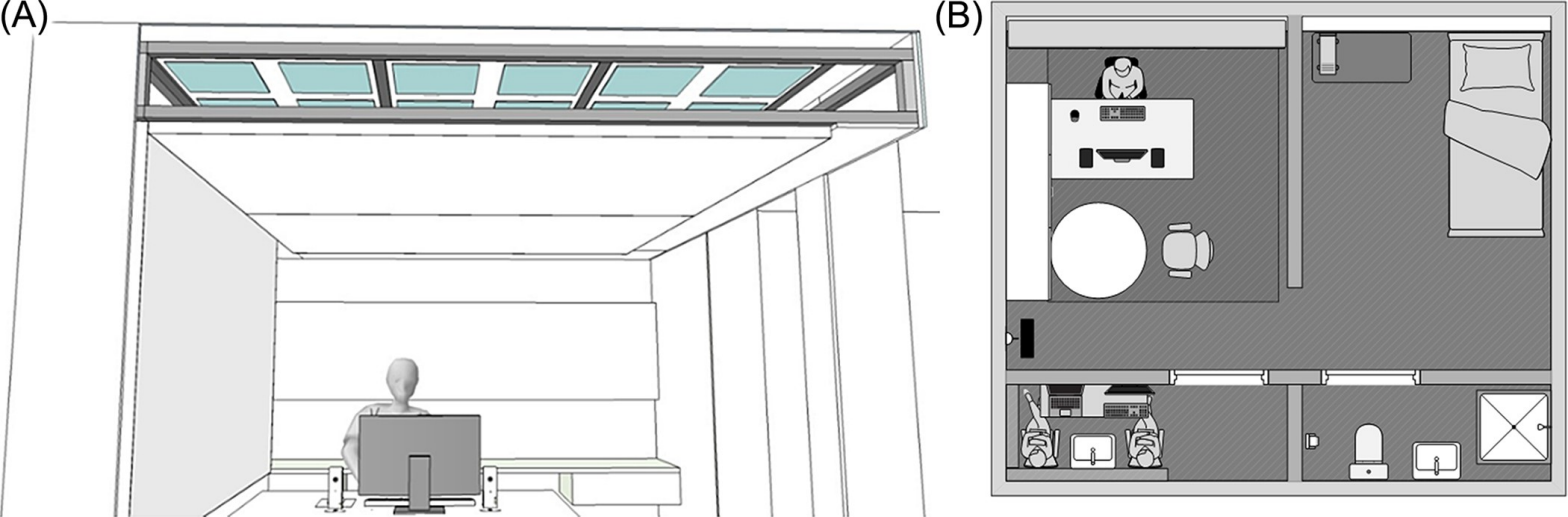
**(A)** A front view of the installed workplace under the Virtual Sky. **(B)** Sketch of the laboratory situation. The participant sits at his workplace and the experimenters are in the anteroom.

The light source (“Virtual Sky”) was a luminous ceiling behind a diffusor foil (Barrisol). The Virtual Sky consisted of a combination of six panels. There were eight LED-tiles placed on every LED-panel measuring 29.2 cm x 29.2 cm. On each tile a combination of 6912 RGB and white LEDs (Daylight White: 5500K; Red: 620–630 nm, Green: 520–535 nm, Blue: 465–475 nm) from LEiDs GmbH & Co. KG was placed. The Virtual Sky could be controlled like a display with a resolution of 48 pixels, each LED tile representing one pixel. To control the light conditions a java program with a socket connection via DMX receiver was used. Sixteen-bit LED drivers (digital multiplex) were installed on the back of the panels and connected to each other in the form of a bus system. Each panel could thus be controlled individually. The conventional pulse width modulation was replaced by a so-called constant current reduction dimming. This has the advantage that undesired effects such as flickering and stroboscopic effects could be avoided. Apart from the luminous ceiling, there was no other light source. Fig 2A) and 2B) show the installed Virtual Sky and the laboratory setting.

### Light settings

SL was planned in accordance with the relevant standards and guidelines for workplace lighting. The standard light at 4000K was used as a baseline setting corresponding to a standard office light. SC and DC were matched in terms of correlated colour temperature and DC in terms of dynamics to daylight. Since the effects of lights’ correlated colour temperature and dynamics could be perceived visually, photopic lux were kept nearly identical for all lighting conditions. Measured vertical illuminance at eye position in 1.30 m height was approximately 300 lx for all lighting conditions. For DC, a minimum illuminance of 175 lx at the eye level was specified (ASR A3.4; [35]). Furthermore, the horizontal illuminance, which was measured at the desk surface was above 500 lx for all lighting conditions, conforming DIN 5034–1 [36]. According to DIN-5035-7, the light colour in an office environment should be warm white (3300 K) or neutral white (3300 K to 5300 K) [29, 37]. The correlated colour temperature under standard lighting was therefore set to 4112 K. The correlated colour temperature of daylight ranges between approximately 5000 and 10000 K [38], and shows a rapidly changing and unstable pattern [39]. SC and DC were adjusted to the average colour temperature of a partly cloudy sky at 8000 K. The colour rendering index (CRI) is a measure of the colour rendering properties of a light source [40]. For an office workstation the CRI should be ≥80 [40]. The median CRI was above 80 for all lighting conditions.

**Fig 3:**
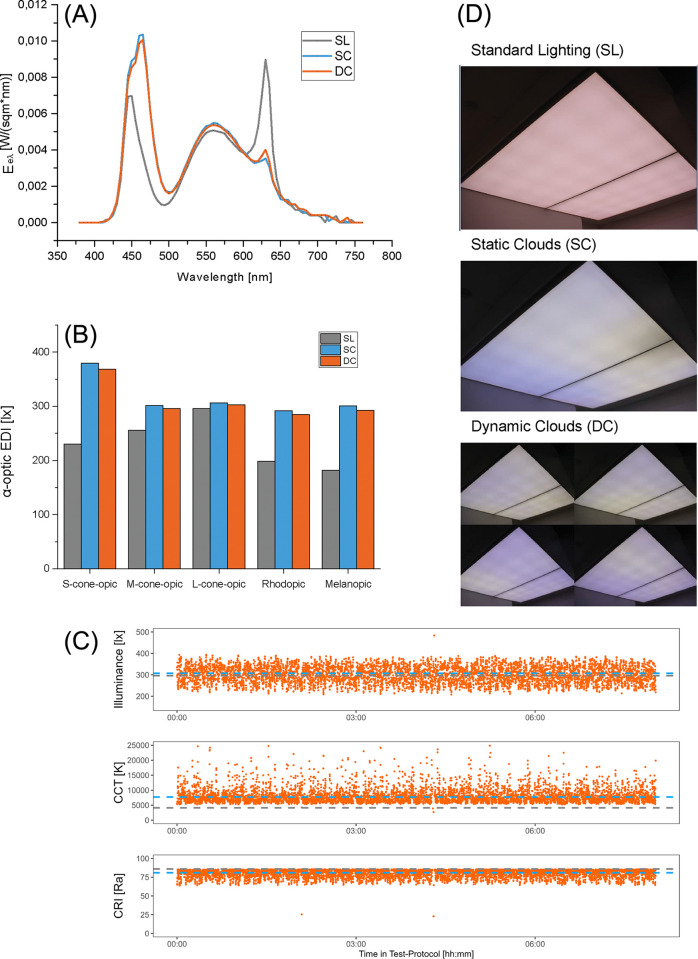
**(A)** Spectral power distribution of the three light conditions (SL, SC, DC). (**B)** Equivalent daylight illuminance of the five different retinal photoreceptor types under SL (grey), SC (blue), and DC (orange). (**C)** Measured colour temperature [K], illuminance [lx] and colour rendering index (CRI) in ~ 5 s intervals during the course of the day. Each point (orange) shows a measurement during DC. Dotted lines show mean values for SL (grey) and SC (blue). (**D)** Photo of the three lighting conditions.

To further examine the variation in DC, we measured the spectral composition of DC in 4 to 5 second intervals over the whole 8 h light exposure. Fig 3A) illustrates the spectral composition and Fig 3B) of the light conditions and equivalent daylight illuminance of the five different retinal photoreceptor types of all three light conditions (see S1 Table in S1 File). Fig 3C shows the in ~ 5 second intervals measured illuminance and colour temperature over time. The dynamic cloud scenario was identical for all participants.

To enable a realistic presentation of the clouds (see Fig 3D), we implemented various setting options in a java program: Intensity of the LEDs, size, contrast, speed, and direction of the clouds and silver-lining effect (the silvery edge of the clouds caused by the sunlight falling on them). The clouds were generated by random noise with a fix seed. The cloud speed was adjusted in such a way that a movement of the clouds could only be perceived by direct observation. Spectral composition was measured using a S-BTS256 spectroradiometer from Gigahertz Optik (Türkenfeld, Germany).

### Test battery procedure

**Fig 4:**
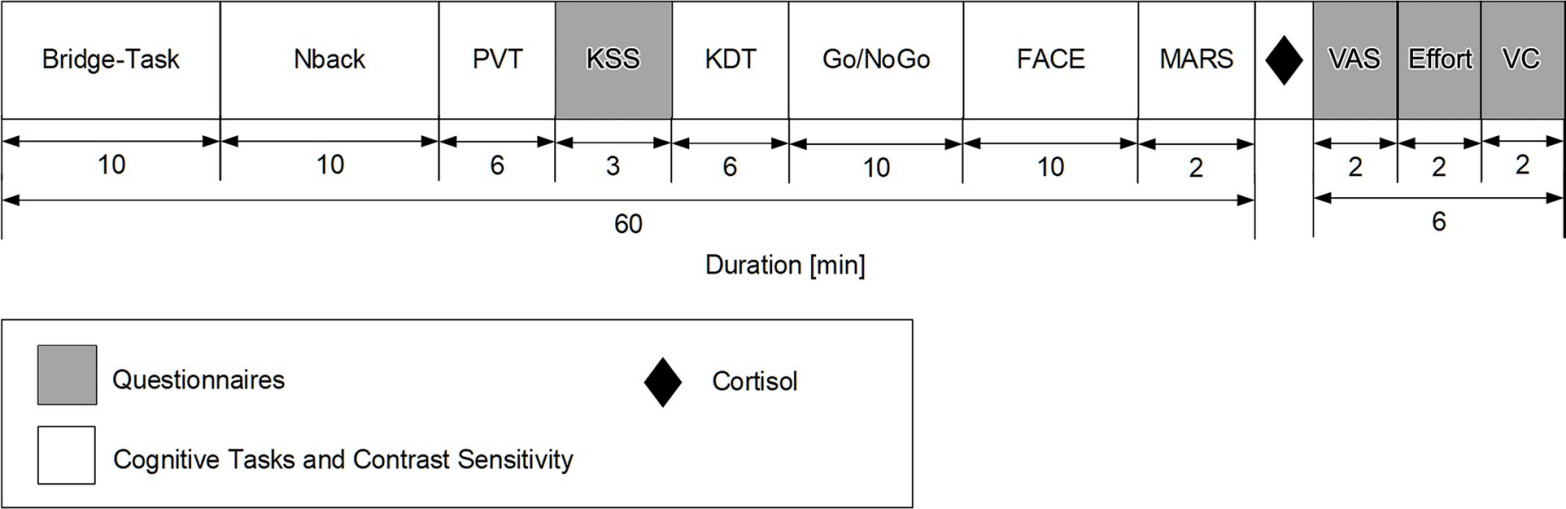
Schematic illustration of one-hour test battery consisting of the Karolinska Drowsiness Test (KDT) and five cognitive tasks: Word-Pair learning task (WPLT), Nback task, Psychomotor Vigilance Task (PVT), Go/NoGo task and Face Emotion recognition task (FACE). After cognitive tasks contrast sensitivity was tested by us of the Mars Letter Contrast Sensitivity Test (MARS). During and after the cognitive tasks several questionnaires were conducted: Karolinska Sleepiness Scale (KSS), Visual Comfort Scale (VCS), Mental Effort Rating Scale (Effort), Subjective Mood and Well-Being (VAS). The Test for Creative Thinking–Drawing Production (TCT-DP) and the Trust Game were only performed once during the afternoon test battery and are therefore not depicted.

During the test sessions in the morning, at midday and in the afternoon, participants performed cognitive tasks and answered questionnaires. Furthermore, contrast sensitivity was tested (see Fig 4).

#### Karolinska Sleepiness Scale (KSS)

Participants rated their sleepiness levels on the Karolinska Sleepiness Scale (KSS [41]) at three different time points during the light exposure. The KSS outcome scale ranges from 1 to 9, every second value is anchored: 1 = “extremely alert”, 3 = “alert”, 5 = “neither alert nor sleepy”, 7 = “sleepy-but no difficulty remaining awake”, 9 = “Extremely sleepy-fighting sleep”.

#### Electroencephalography (EEG)

EEG activity was recorded from 21 Ag/AgCl active electrodes placed in a cap (actiCap, Brain Products, Munich) based on the 10–20 system (Fp1, Fp2, F7, F3, Fz, F4, F8, T7, C3, Cz, C4, T8, P7, P3, Pz, P4, P8, M1, M2, O1, O2). The recording bandwidth was 0.3 to 70 Hz. Data were sampled at 500 Hz using a LiveAmp wireless EEG-amplifier system (Brain Products, Munich). During recording, all electrodes were referenced to AFz; for data analysis, they were re-referenced to the linked mastoids (mean of M1 and M2). Vertical and horizontal eye movements were recorded from electrodes placed laterally to both eyes and above and below the left eye (EOG). Averaging and artifact rejection was performed off-line.

#### Karolinska Drowsiness Test (KDT)

The Karolinska Drowsiness Test (KDT) was used as an objective index of sleepiness and the concomitant impairment on alertness and vigilance [41]. Subjects were instructed to relax, sitting in a comfortable, supine position and fixate a point on the wall at a distance of 1 m for 4 minutes, while EEG and EOG were registered. EEG recordings were processed by using the BrainVision Analyzer software (Version 2.1.1; Brain Products GmbH, Gilching, Germany). Ocular artefacts were removed applying an independent component analysis approach, then the signals were filtered (Butterworth filters: low cut-off 0.5 Hz, order 8; high cut-off 30 Hz, order 8; notch filter: 50 Hz). Subsequently, recordings were segmented into 120 epochs of 2 s. Epochs with artefacts were automatically removed by software (amplitude criterion ±100 μV) and the remaining epochs were visually inspected for residual artefacts; at least 80% of the epochs were valid across all 4-min segments. Absolute as well as relative EEG power spectra were computed by fast Fourier transformation on the 2-s epochs, resulting in spectrograms from 0.5 to 250 Hz with a resolution of 0.5 Hz.

Derivations of the four major regions were combined for the analyses: frontal F (F3, Fz, F4), central C (C3, Cz, C4), parietal P (P3, Pz, P4), and occipital O (O1, O2). The epoch power spectra were averaged per derivation area (F, C, P, O) for each testing. Alpha band power (8 Hz– 12 Hz) was integrated using Simpson’s rule, a polynomial based approximation technique.

#### Psychomotor Vigilance Task (PVT)

Sustained attention performance was assessed using the Psychomotor Vigilance Test approach of Dinges, which has demonstrated sensitivity to circadian variation and sleep loss [42]. It involves a visual reaction time (RT) performance test of five minutes duration. A timer, counting from 0 to 1000 in white numbers, appeared on a previously black screen at random inter-trial intervals between 1 and 10 seconds. When the timer appeared, subjects had to press the space bar as fast as possible to stop the timer. PVT performance measure was the median response time (RT) of correct responses within a time window between 180 ms and 500 ms.

#### Go/NoGo

A Go/NoGo paradigm (implemented in *Presentation*, v16, Neurobehavioral Systems, Inc., Berkley, CA, USA) was used to measure sustained attention and response inhibition. The task was to respond as fast as possible to only one of two visual stimuli (Go condition) by pressing the space bar on a computer keyboard. The visual stimuli were the letters “M” (Go condition, 80% of trials) and “W” (NoGo condition, 20% of trials). Responses to NoGo stimuli were counted as commission errors, and failures to respond to Go stimuli within 180 to 500 ms after stimulus onset were counted as omission errors. The white letters were presented in random sequence on a computer screen with black background at 1 m viewing distance. Four hundred trials were conducted in approximately 10 minutes with an average interstimulus interval of 1.500 ms (± 200 ms). The rationale of this paradigm is to habituate the Go response to the frequent stimulus “M” and to measure the ability to inhibit the response to the rarer NoGo stimulus “W”. The percentages of omission and commission errors were analyzed.

#### Word-Pair Learning Task (WPLT)

Declarative memory performance was tested via a Word Pair Learning Task which consists of pairs of semantically unrelated words. The WPLT previously used by Cajochen et al (2011) was shortened to 40 word pairs [43]. During the encoding session, each word pair was displayed on the screen for 6 s, followed by a white, centred fixation cross for 5 s. Participants were instructed to visually imagine a relationship between the two paired words. Immediately after the encoding session, a recognition task was performed. Twenty previously studied word-pairs (old), in addition to 20 newly arranged word-pairs which were also built from previously learned words, were presented in mixed order. For each word pair, the participant had to indicated whether the pair had been presented before (old), likely presented before, or newly arranged. The classification had to be made within 4.5 seconds for a trial to be valid. For the 9 test batteries (3 applications per light condition) 9 different encoding and recall word lists were created and assigned in a pseudo-randomized order. The Discrimination Index (*d’*, “*d* prime”) is a measure of recognition memory strength based on signal detection theory. It was calculated from the correctly remembered old word-pairs (hits) and the new word-pairs that were incorrectly classified as old word-pairs (false alarms) [44]. The assessment of declarative memory performance was based on the percentage of correctly remembered old word pairs correctly identified rearranged word pairs [43], and the Discrimination Index (*d*’, “*d* prime”) [44].

#### N-back test

The ability of the participants to manipulate information stored in working memory was assessed with an n-back task (n-back levels 0, 2 or 3) [45]. Stimuli consisted of pseudo-randomized sequences of phonologically dissimilar consonants (F, J, H, K, L, M, R, S, T, R). Subjects had to indicate by button press whether the displayed letter matched the stimulus presented n trials before (n-back level 2 or 3). In the 0-back condition, they had to indicate whether the current stimulus matched the predetermined letter “K.” In all three conditions, targets were presented in 33% of the trials. Each session consisted of five blocks for each of the three n-back conditions (15 blocks in total) presented in pseudorandomized order (maximum of two blocks of the same condition in succession). Each block consisted of 33 trials (stimulus duration: 500 ms; inter-stimulus interval: 1.000 ms, response time 1.000 ms). Before each block, a cue appeared indicating the condition to be performed. Error rate (%) of all three n-nback conditions was analyzed.

#### Trust Game

The Trust Game was used to study the influence of the different light conditions on trust dispositions for decision making in transactional situations [46]. The game was realized as an application running in a web-browser window; input was given by mouse clicks on buttons. The players were endowed with 10 Swiss Francs (CHF), represented as a stack of coins on the display, and were given a single chance to transfer an amount of their choice from their endowment to a second (anonymous) player via the web interface. In the Trust-Game participants were informed that the second player would receive triple the amount of the transferred money and could but was not obliged to return any amount back to them, and that the resulting amount was actually paid out. No additional information about the other player was revealed. The game is usually a two-person interaction, but for the current experiment the second person was in fact a computer-generated response, which always returned 150% of the invested amount to the player. Thus, he might maintain the Nash equilibrium [47] by not sending any amount or engage to an individual degree in the transaction with uncertain outcome, depending on his trust propensity. The Trust Game was played only once under each light condition, each player’s invested CHF stake value entered the analysis as indicator for trust disposition.

#### Test for Creative Thinking–Drawing Production (TCT-DP)

For evaluating the creative potential under the different lighting conditions, Urban’s (2004) drawing test was used [48], it was applied once in the afternoon of each experimental day. Participants had to continue drawing on a paper with few given basic lines and shapes. Since there are just two available models, version C was created by 90° rotation of version A. The drawing production was evaluated by means of a set of 14 key evaluation criteria resulting in a scale value between 0 and 72.

#### Mental effort rating scale

Participants rated perceived given effort, effort relative to previous test-battery, performance self-satisfaction, focusing effort, exhaustion and motivation during the test-battery directly after its completion by use of visual analogue scales (VAS) with the extreme values “low” and “very” [49]. Subjects’ markings on the VAS were interpreted as percentage relative to the maximum extreme of the scale.

#### Subjective mood and well-being

VAS were also used to assess current tension, physical comfort, fatigue, sleepiness and mood [50]; the response was interpreted as percentage of maximum scale expression.

#### Salivary cortisol

Saliva samples were collected in hourly intervals during the course of the day under all three light conditions. The first saliva sample was taken in the morning at 9:00 a.m., immediately after the start of the respective light exposure. The last saliva sample was collected towards the end of the test day at 5:00 p.m. The preparation of the cortisol saliva samples was carried out in the in-house laboratory of the University Psychiatric Clinics in Basel. Prior to the measurement of cortisol, the saliva sample was vortexed and centrifuged at 1500 x g for 15 minutes to remove the precipitate. According to the manufacturer’s protocol, the cortisol concentration was determined using the high-sensitivity salivary cortisol enzyme immunoassay kit (Salimetrics Europe, UK) [51, 52]. The optical density was detected at 450 nm using the Cytation 3 Cell Imaging Multimode Reader (BioTek). The assay is a competitive immunoassay where the detected signal is inversely proportional to the amount of cortisol present in the sample. The cortisol ELISA had a sensitivity of <0.007 ug/dL, an intra-assay CV of 4.6% and an inter-assay CV of 6%.

#### Visual Comfort Scale (VCS)

To assess each participant’s subjective perception of visual comfort, we used a five-point Likert type scale that probed acceptance of light situation, brightness, light colour, and glare perception, influence of light on alertness and on focus, based on a selection of questions derived from Eklund and Boyce [53]. Except for the perceived glare scale, which was interpreted as unipolar on a scale from 1 to 5, the responses were mapped to the bi-directional range from -2 to +2.

#### Contrast sensitivity (MARS)

The Mars Letter Contrast Sensitivity Test (MARS) consists of three panels, each with 48 letters printed on it with decreasing contrast [54]. The letters C, D, H, K, N O, R, S, V and Z are used. The contrast of the successive letters decreases by a constant factor of 0.04 logarithmic units. The logarithmic contrast sensitivity can thus be tested from 0.04 (contrast: 0.912) to 1.92 (contrast: 0.012). In this study, contrast sensitivity was measured monocularly. The non-tested eye was covered with a standard lightproof eye patch. The leading or non-leading eye was examined in a counter-balanced, pseudorandomized order. The charts were placed in front of the test subjects at a distance of 0.5 m. The participant was instructed to read the letters from left to right, from top to bottom. The measurements were carried out according to a forced choice strategy, whereby the test person was encouraged to guess. The contrast sensitivity corresponds to the last letter before two consecutive letters were not correctly named, minus the letters that were not correctly named before.

### Statistical analysis

The primary outcome of this study was to elucidate whether the three light conditions affected cognitive performance and the subjective state of mood and well-being. Of further interest was how the outcome variables, measured three times on each treatment day, evolved throughout the assessment under each light condition, and if the light condition would influence their course. We performed univariate linear mixed model (LMM) analysis for each dependent variable (DV), and a survey of all outcome variables is given in S2 Table in S1 File. The general design of the LMMs comprised two fully crossed repeated measures factors, factor *Light Condition* (*LC*) with three levels coding for the type of light exposure throughout an assessment day: *standard light* (*SL*), *static cloud* (*SC*), and *dynamic cloud* (*DC*), as well as factor *Time of Day* (*ToD*) indicating the point of measurement on an assessment day (*morning*, *midday*, and *afternoon*). The repeated measures per subject were modelled as individual random intercept effect.

With each dependent variable four alternative models were constructed: 1) the fully crossed model with *Light Condition* and *Time of Day* as fixed effects including their interaction, 2) the additive two-factorial model with fixed effects *Light Condition* and *Time of Day* without interaction, and 3 & 4) the one-factor models with *Light Condition* or *Time of Day*, each as fixed effect. From these four alternatives the best model was selected by the following scheme: if a significant interaction effect in the fully crossed model was present, that model was selected; otherwise the model with the lowest Akaike Information Criterion (AIC) was selected among the other three models. Contrast testing was done for significant fixed effects in the selected model only.

Statistical analyses were performed using the R statistical computing environment (Version 4.0.3, [55]. The R packages used for statistical analysis can be found in the S1 File. LMM fixed effects were tested for significance by one- or two-factorial analysis of deviance (AoD) Wald-type chi squared (Χ^2^) statistics. Cohen’s omega squared (ω^2^) is provided as effect size measure for the LMM fixed effects; it can be interpreted as follows: very small effect: ω^2^ < .01, small effect: ω^2^ ≥ .01, medium effect: ω^2^ ≥ .06, large effect: ω^2^ ≥ .14 (analogous to eta squared, [56]); for models with more than one fixed effect, partial omega squared (ω_p_^2^) is reported, the same interpretation applies. Contrast testing of significant AoD fixed effects was performed using Šidák or Tukey adjustment for multiple testing. To clarify the nature of significant interaction effects, the simple main effects were calculated, which is the pairwise comparison of all levels of one factor, done separately for the levels of the second factor, e.g. comparing all lighting conditions with each other, but separately for Morning, Midday, and Afternoon. Leaving out non-interesting contrasts/comparisons reduces the number of tests conducted, in our case from 36 to 18 pairwise comparisons. Effect size for comparisons between conditions are given as Hedges’ *g* (very small effect: *g* < .20, small effect: *g* ≥ .20, medium effect: *g* ≥ .50, large effect: *g* ≥ .80, analogous to Cohen’s *d*, 1988). A *p*-value < .05 was considered indicating statistical significance, however, for the entirety of selected LMMs, a *q*-value based false discovery rate analysis (FDR; Storey, 2002 [57]) was performed and significant effects above an FDR limit of 5% were reported, but excluded from post-hoc evaluation and discussion.

For the procedure with unmet LMM assumptions of homoscedasticity and homogeneity of variances see S1 File.

Results and figures report back-transformed response scale values.

Eleven data points of six participants, which indicated that participants did not comply with task instructions, i.e. if error rates were inconsistently over 90% or the change within a light condition exceeded 25%, were removed from the data set prior to analysis, no further outliers were identified and no missing data imputation was performed.

*Salivary Cortisol*: In order to test the hypothesis that the fall-off rate of cortisol during morning hours may be influenced by the light setting, in a first step, the decay constant of a three parameters asymptotic regression model (*SSasymp*, three parameters: intercept, lower asymptote, and decay rate constant) of the four morning cortisol samples on the time of measurement was estimated. The parameter estimates per subject per light condition were derived by fitting a non-linear mixed model (NLMM) to the untransformed salivary cortisol data. In a second step, the decay rate constant parameter estimates were compared between the light settings by a one-factorial LMM approach with fixed effect *Light Condition*. Additionally, salivary cortisol AUCs for the morning hours 9:00 h through 12:00 h were calculated for each subject using the trapezoid method and were compared by one-factorial LMM between the three light conditions as well. Due to strong spikes of individual cortisol levels during lunch time, the samples after 12:00 p.m. were not considered in the regression model.

### Results

An FDR analysis of the *p*-values for a total of 81 selected LMM effects was performed. The highest *q*-value indicated an FDR of 10% among the 18 tests that were significant with *p* < .05, see S9 Table in S1 File. Seven significant effects with *q* > 0.05 were omitted from post-hoc evaluation and discussion.

### Descriptives

The average age of the 18 enrolled male subjects was 25.17 years (SD = 3.75 years), they all indicated that they are right-handed, and 50% of them were right-eye dominant (Rosenbach Test, [58]). General sleep quality was good at enrollment as the low score in the Pittsburgh Sleep Questionnaire indicated (mean score 3.17, SD = 1.20). The average time was 4:38 (h:min) for Midsleep on Free Days corrected (Sleep time on free days corrected for sleep duration on workdays (MSFsc) range 2.7–6.4, SD = 0.97), which corresponds to an intermediate chronotype.

### Alertness

#### Subjective Sleepiness (KSS) and Momentary Sleepiness (VAS)

The KSS sleepiness ratings were rather stable across the entire day under all three light conditions, there were no significant effects; all *p* > .19 (S4 Table in S1 File). Allover *Subjective Momentary Sleepiness* as measured by VAS, showed a marginally significant *Time of Day* effect above the FDR threshold (*Momentary Sleepiness*, one-factorial AoD of *ToD*: Χ^2^_(2)_ = 6.15, *p* < .046, ω^2^ = .01, small effect). This effect was not modulated by light condition, *p* = .54.

#### Objective sleepiness (KDT)

Objective Sleepiness was measured in the wake EEG by analysing *Alpha Band Power*. *Alpha Band Power* yielded no significant *Light Condition* main effect in any of the four cranial areas; all *p* >.28.

**Fig 5:**
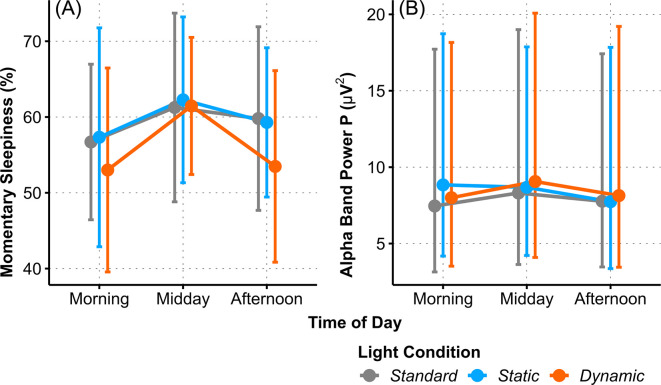
Interaction plots of estimated means of (A) Momentary Sleepiness Ratings (VAS, %) and (B) KDT *Alpha Band Power P*. Error bars indicate adjusted 95% confidence intervals.

Only in the parietal region P, some small and very small interaction effects of *Light Condition* with *Time of Day* were observed, however, this interaction effect was only marginally significant and was omitted due to the FDR limit (*Alpha Band Power P*, *LC* × *ToD*: Χ^2^_(4)_ = 9.69, *p* = .046, ω_p_^2^ = -.01, very small). KDT outcomes for other cranial regions showed significant but mainly very small *Time of Day* effects, for instance *Alpha Band Power C* (*ToD*: Χ^2^_(2)_ = 9.14, *p* = .01, ω_p_^2^ = .02, small), indicating a significant rise from *morning* to *midday* and reduction in the *afternoon* (*morning–midday*: *t*_(136)_ = 2.75, *p* = .02, *g* = 0.17, very small; *midday–afternoon*: *t*_(136)_ = 2.72, *p* = .02, *g* = 0.11, very small). For a summary of these we refer to S3 Table and S4 Fig in S1 File. In conclusion, only for the DC condition the increase in subjective sleepiness from morning to midday was accompanied by some general, significant increase of objective sleepiness (Fig 5B).

### Cognitive performance

#### Psychomotor Vigilance Test (PVT) and working memory (N-back)

Neither response time nor the percentage of errors of the Psychomotor Vigilance Test (PVT) or any of the N-Back Test variants yielded any statistically significant effects of *Light Condition*, *Time of Day* or their interaction; all *p* > .09 (see S5 Table in S1 File).

#### Word-Pair Learning Task (WPLT)

Neither light conditions nor time of day showed any significant effects on the percentages of correctly identified old word-pairs, newly rearranged word-pairs, or the rate of incorrectly classified word-pairs, all *p* >.06 (see S5 Table in S1 File).

#### Go/NoGo

**Fig 6:**
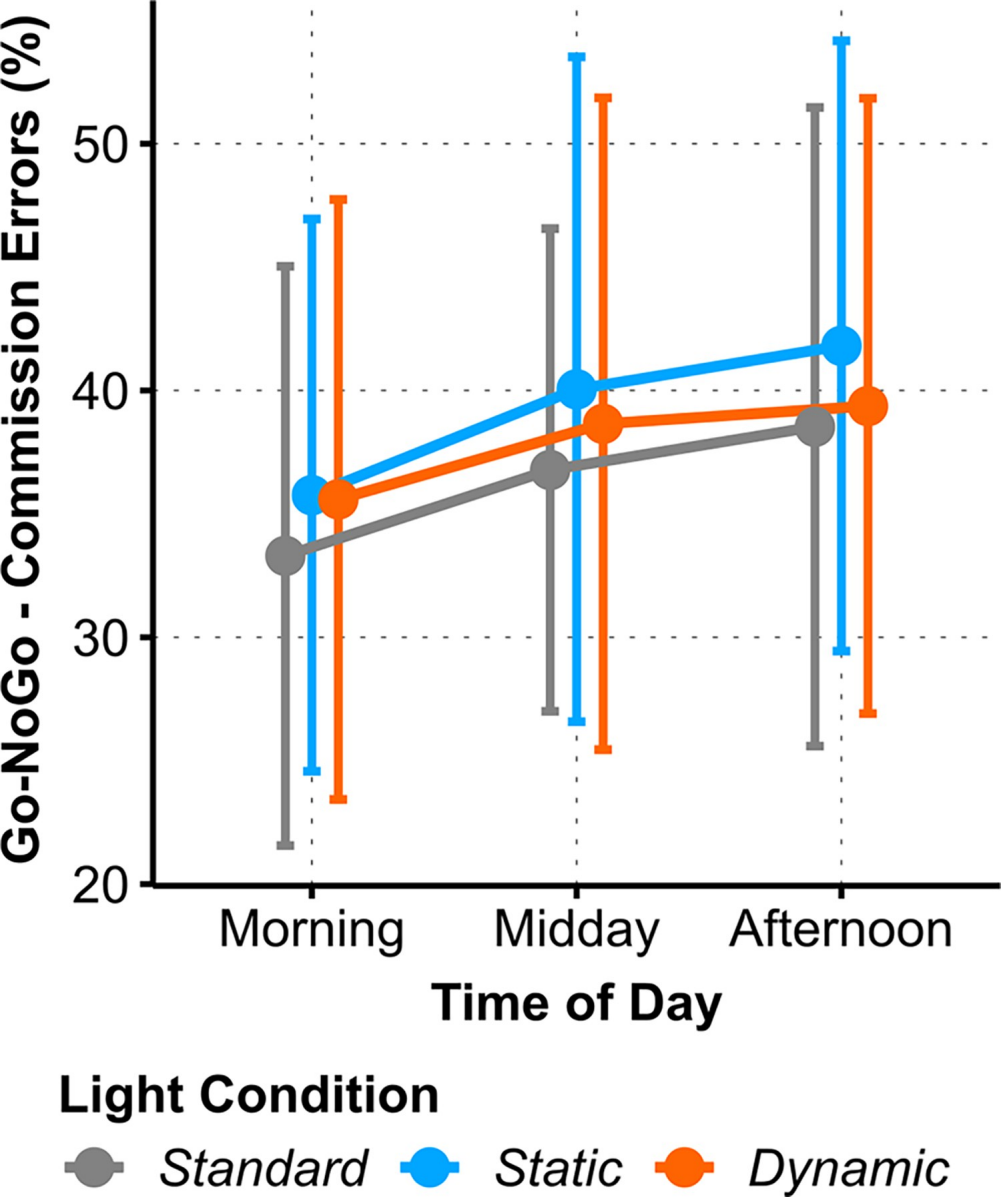
Interaction plots of estimated means for Go-NoGo commission errors (%) between *Light Conditions* across *Time of Day*. Light condition did not modulate the increase of commission errors over day. Error bars indicate adjusted 95% confidence intervals.

The percentages of commission and omission errors of the Go-NoGo task were analyzed. *Commission Error* rate did not significantly differ between the light conditions, but showed a marginally significant *Time of Day* effect (one-way AoD of *ToD*: Χ^2^_(2)_ = 6.21, *p* = .045, ω^2^ = .04, small), which was eliminated due to the FDR limit (Fig 6). For *Omission Errors*, no significant effects were found; all *p* > .07 (see S5 Table in S1 File).

#### Test for Creative Thinking–Drawing Production (TCT-DP) & Trust Game

Neither creativity (*p* = .86) nor trust propensity (*p* = .20) were influenced by the light conditions, see S6 Table in S1 File.

### Subjective effort ratings for the cognitive tasks (RSME)

Among the six subscales of the Mental Effort Rating Scale (RSME) four of them showed small and medium sized effects (S7 Table in S1 File).

**Fig 7:**
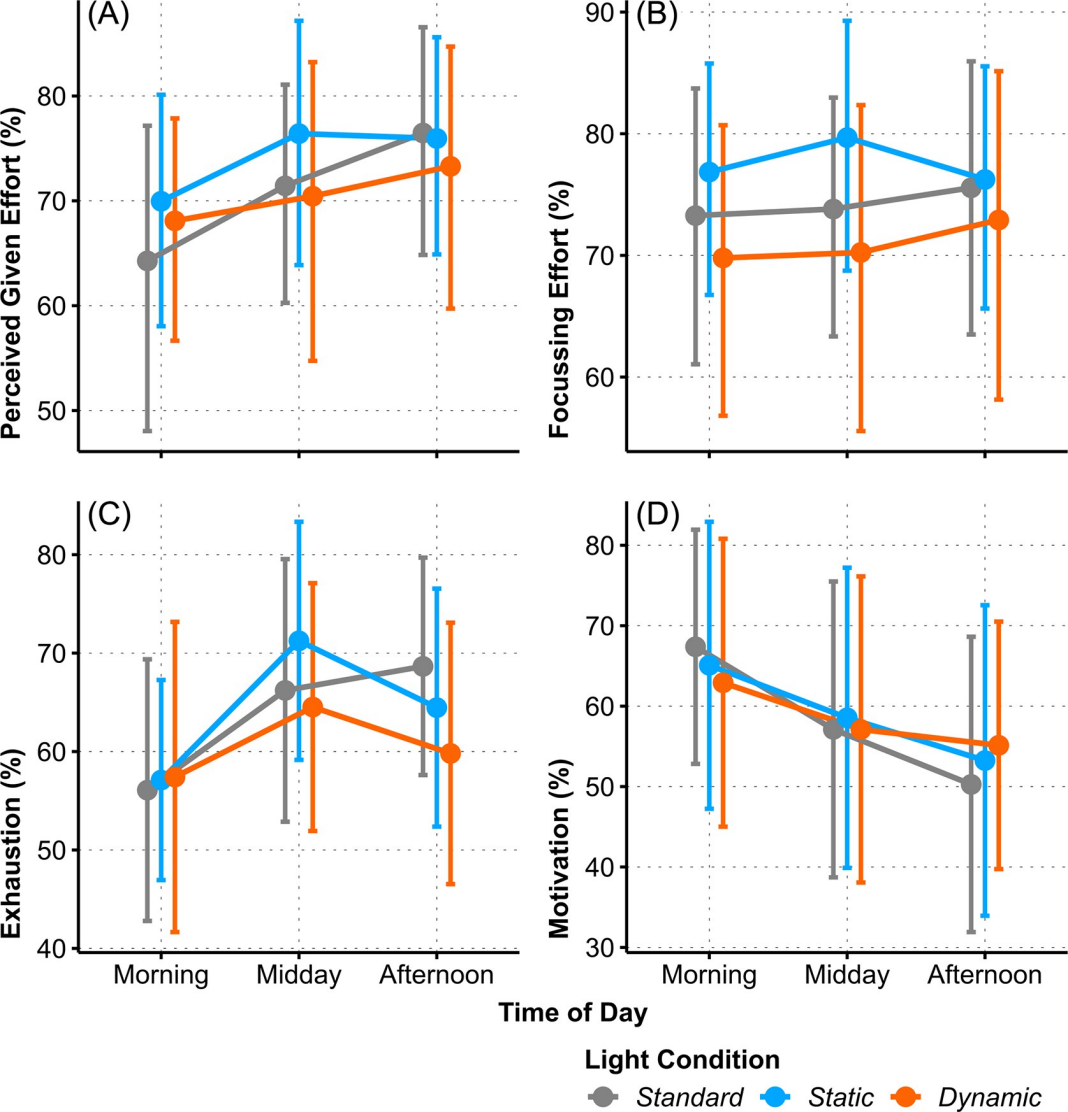
Interaction plots of estimated means for the Mental Effort Rating Scales: (A) *Perceived Given Effort* (%), (B) *Focussing Effort* (%), (C) *Exhaustion* (%), and (D) *Motivation* (%) between *Light Conditions* across *Time of Day*. Error bars indicate adjusted 95% confidence intervals.

The perceived *Focussing Effort* during the cognitive tasks varied depending on *Light Condition* (one-factorial AoD of *LC*: Χ^2^_(2)_ = 12.71, *p* = .002, ω^2^ = .08, medium). It was rated highest in the *SC* condition and lowest in the *DC* condition, the significant effect occurred between these extremes (*SC–DC*: *t*_(142)_ = 3.41, *p* = .002, *g* = 0.26, small), see Fig 7B); no *Time of Day* effect was observed.

The other three RSME scales *Exhaustion*, *Perceived Given Effort*, and *Motivation* evolved across *Time of Day* and were not influenced by the light setting: The level of rated *Exhaustion* increased from morning to midday (one-factorial AoD of *ToD*: Χ^2^_(2)_ = 7.18, *p* = .03, ω^2^ = .06, small), but the effect was removed from consideration due to the FDR limit. Ratings of *Perceived Given Effort* and *Motivation* developed monotonously throughout the day, see Fig 7A) and 7D), respectively. *Perceived Given Effort* significantly increased from morning to midday and remained elevated (one-factorial AoD of *ToD*: Χ^2^_(2)_ = 9.08, *p* = .01, ω^2^ = .07, medium; *morning–midday*: *t*_(142)_ = 2.67, *p* = .02, *g* = 0.21, small; *morning–afternoon*: *t*_(142)_ = 2.79, *p* = .02, *g* = 0.31, small). Rated *Motivation* described a trajectory in the opposite direction, while being highest in the morning it continuously declined towards the afternoon (one-factorial AoD of *ToD*: Χ^2^_(2)_ = 31.36, *p* < .001, ω^2^ = .12, medium; *morning–afternoon*: *t*_(141)_ = 5.50, *p* < .001, *g* = 0.32, small). No effects were found for the RSME subscales *Satisfaction with Performance* and *Effort Relative to Previous Testing* (see S7 Table in S1 File).

### Subjective mood and well-being

**Fig 8:**
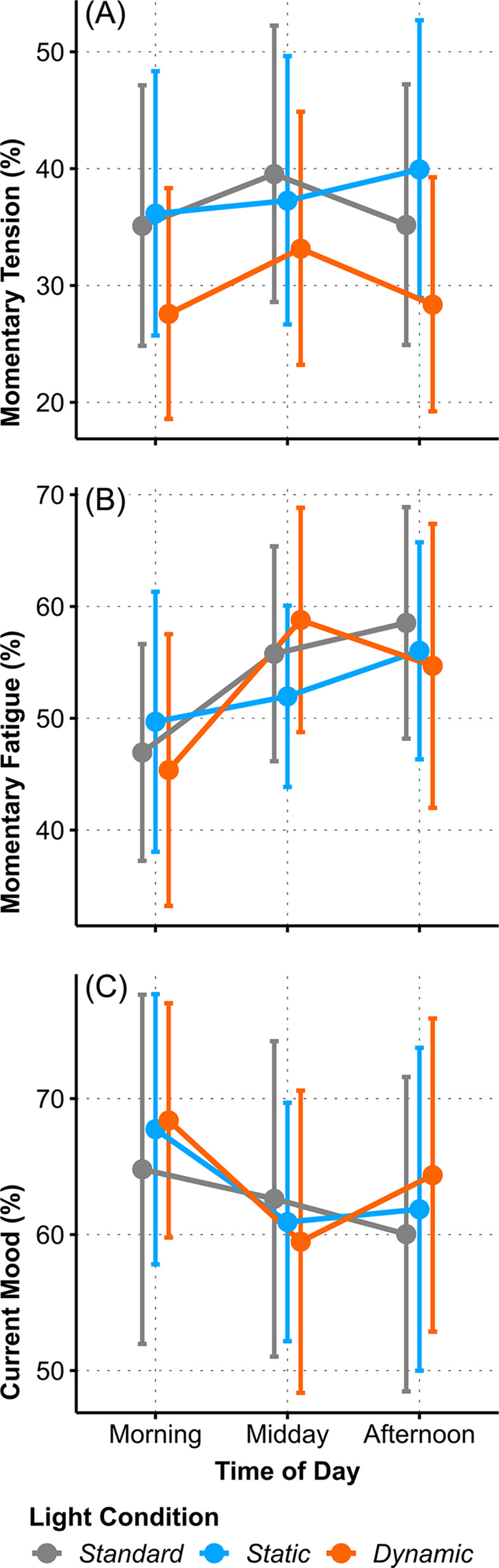
Interaction plots of estimated means for the mood and state ratings. (A) *Momentary Tension* (%), (B) *Momentary Fatigue* (%), and (C) *Current Mood* (%) between *Light Conditions* across *Time of Day*. Error bars indicate adjusted 95% confidence intervals.

Amid the subjective ratings of mood and well-being only *Momentary Tension* was influenced by *Light Condition* (one-factorial AoD of *LC*: Χ^2^_(2)_ = 9.76, *p* = .008, ω^2^ = .05, small). The rating was similar in the *SL* and *SC* setting and in both of them significantly higher than in the *DC Light Condition* (*SL–DC*: *t*_(142)_ = 2.48, *p* = .04, *g* = 0.36, small; *SC–DC*: *t*_(142)_ = 2.88, *p* = .01, *g* = 0.42, small), see Fig 8A). Significant changes throughout the day were registered for *Momentary Fatigue*, and *Current Mood* (*Momentary Fatigue*, one-factorial AoD of *ToD*: Χ^2^_(2)_ = 13.41, *p* = .001, ω^2^ = .07, medium; *Current Mood*, one-factorial AoD of *ToD*: Χ^2^_(2)_ = 17.93, *p* < .001, ω^2^ = .04, small), see Fig 8B), and 8C).

Subjective *Momentary Fatigue* and *Current Mood* described antagonistic patterns during the day. *Momentary Fatigue* was rated lowest in the morning and increased significantly towards midday; it remained on that elevated level in the afternoon (*Momentary Fatigue*: *morning–midday*: *t*_(142)_ = 3.63, *p* = .001, *g* = 0.49, small; *morning–afternoon*: *t*_(142)_ = 2.86, *p* = .01, *g* = 0.44, small). *Mood* was rated highest in the morning, dropped significantly towards midday (*Current Mood*: *morning–midday*: *t*_(142)_ = 4.18, *p* < .001, *g* = 0.28, small). Perception of *Physical Comfort* was not altered by any of the light conditions, nor did it fluctuate by *Time of Day*, all *p* > .2 (see S4 Table in S1 File).

### Salivary cortisol

**Fig 9:**
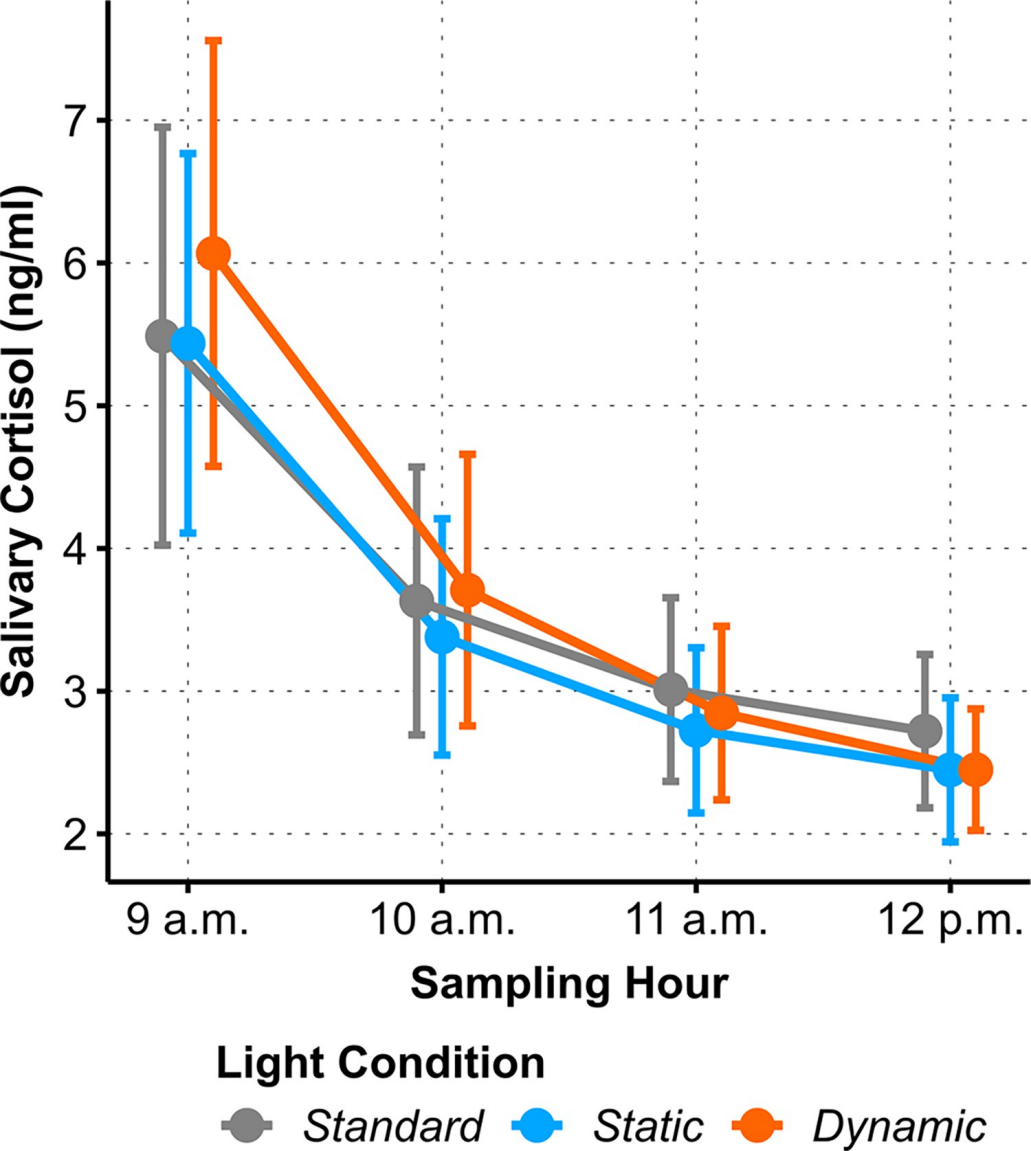
Averaged asymptotic regression NLMM predictions of salivary cortisol to the four morning hours for each of the three *Light Conditions*. Error bars show 95% confidence boundaries.

The LMM analysis of the salivary cortisol decay parameter during the four morning hours did not reveal differential dynamics between the light conditions (*p* = .44), see Fig 9. Furthermore, salivary cortisol AUC in the morning hours did not reveal any light conditions differences.

### Visual comfort and contrast sensitivity

#### Visual Comfort Scale (VCS)

**Fig 10:**
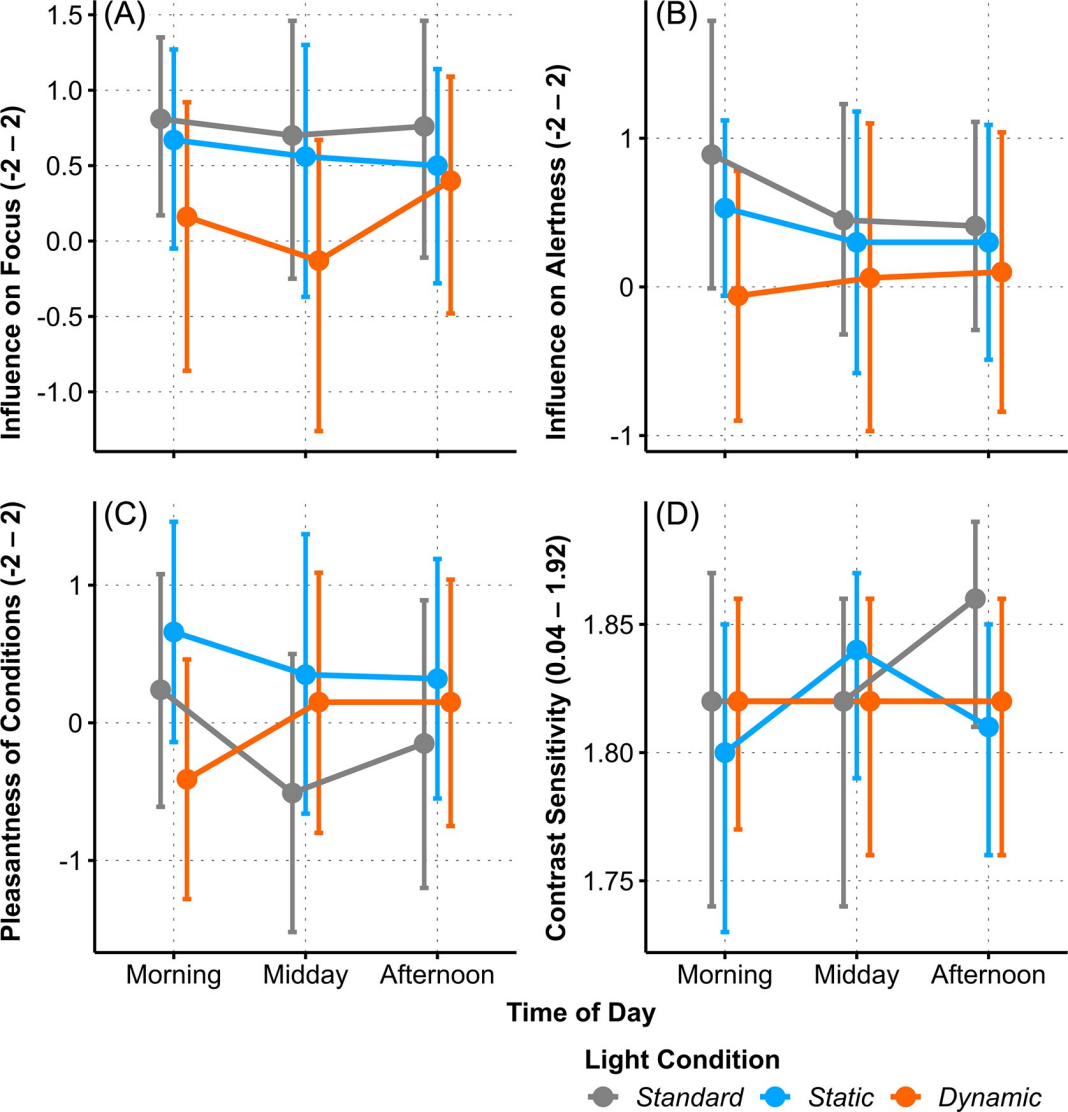
Interaction plots of estimated means for Visual Comfort Ratings: (A) *Influence on Focus*, (B) *Influence on Alertness*, (C) *Pleasantness of Conditions*, and (D) *Contrast Sensitivity* between *Light Conditions* across *Time of Day*. Error bars indicate adjusted 95% confidence intervals.

The light of the three conditions was perceived differently regarding three of the six Visual Comfort aspects measured by the VCS. Subjects attributed a significantly higher positive *Influence on Focus* to the *SL* and *SC* settings than to the *DC* condition (one-factorial AoD of *LC*: Χ^2^_(2)_ = 10.53, *p* = .005, ω^2^ = .15, large; *SL–DC*: *t*_(77)_ = 2.43, *p* = .045, *g* = 0.36, small; *SC–DC*: *t*_(77)_ = 2.71, *p* = .02, *g* = 0.23, small), compare Fig 10A). There was no significant impact of *Time of Day*. The perceived *Influence on Alertness* occurred modulated by both *Light Condition* and *Time of Day* (two-factorial AoD, *LC* × *ToD*: Χ^2^_(4)_ = 9.66, *p* = .047, ω_p_^2^ < .001, very small), but this marginally significant effect fell below the FDR limit.

The perceived *Pleasantness of Conditions* depended on both *Light Condition* and *Time of Day* (two-factorial AoD, *LC* × *ToD*: Χ^2^_(4)_ = 14.92, *p* = .005, ω^2^ = .08, medium). Specifically, in the morning it was rated highest for *SC*, whereas the *DC* condition was perceived significantly less pleasant (*morning*, *SC–DC*: *t*_(83)_ = 3.42, *p* = .003, *g* = 0.87, large), as shown in Fig 10C.

There was no significant difference of rated *Brightness*, *Colour and Glare of Conditions*, between the light conditions, nor across time of day (see S8 Table in S1 File).

#### Contrast sensitivity (MARS)

Contrast Sensitivity presented a *Light Conditions* × *Time of Day* interaction effect (two-factorial AoD, *LC* × *ToD*: Χ^2^_(4)_ = 37.08, *p* < .001, ω_p_^2^ = .05, small). Among *SC* and *DC* light settings, sensitivity was measured similar throughout the day, also the estimated mean values under *SL* were not different from *SC* and *DC*, compare Fig 10D). However, in the *SL* setting the *afternoon* measurement was significantly higher than those of *morning* and *midday*, as well as significantly higher than the *afternoon* measures of *SC* and *DC* (*SL*, *morning–afternoon*: *t*_(136)_ = 3.04, *p* = .008, *g* = 0.57, medium; *SL*, *midday–afternoon*: *t*_(136)_ = 3.99, *p* < .001, *g* = 0.60, medium; *afternoon*, *SL*–*SC*: *t*_(136)_ = 3.03, *p* = .008, *g* = 0.72, medium; *afternoon*, *SL*–*DC*: *t*_(136)_ = 2.72, *p* = .02, *g* = 0.65, medium).

### Discussion

The aim of this study was to investigate whether an eight-hour exposure to three different light scenarios (SL, SC, and DC) unfolds differential effects on subjective and objective correlates of sleepiness and stress levels, well-being, visual and cognitive performance, and creativity during office work conditions.

### Can nature-adapted lighting keep us alert?

We could not observe a significant effect of nature adapted lighting on subjective alertness and psychomotor vigilance. Although subjective alertness and psychomotor vigilance were rather stable over the course of the day, subjective fatigue and effort were higher at midday compared to morning. Interestingly, volunteers rated their concentration effort during the cognitive tasks higher during SL compared to DC, which might indicate that the effort for completing the tasks was reduced during dynamic lighting without affecting performance measures. The same type of light source, but in the form of a light wall, was used by Lasauskaite et al. (2018, 2023). They could show that that working in light conditions that differ in correlated colour temperature (2800–6500 K) required a higher amount of effort (i.e. effort-related cardiac response, indexed by a shortened cardiac pre-ejection period) in lower compared to higher correlated colour temperatures. In line with our study results, they could not find effects on cognitive performance or sleepiness. However, we found a positive effect on subjective concentration effort when increasing CCT and presenting dynamic clouds while increasing CCT alone was not enough to affect subjective correlates of subjective concentration effort [59, 60]. In contrast to the latter, several studies reported alerting effects of monochromatic blue-light or higher CCTs (i.e.>4000 Kelvin) during the day [61–64]. Moreover, Stefani et al. (2012) showed reduced afternoon sleepiness during the presentation of a cloud simulation compared to static office lighting [20].

During the day, only a few studies have observed light effects on objective sleepiness as measured by waking EEG [13, 14, 61, 65–67]. Zeeuw et al. reported that reducing melanopic radiance resulted in an increase in EEG alpha activity at a rather low illuminance (100 lx). Under similar illuminance levels (between 200 and 1200 lx), as ours (~ 300 lx), they could only observe decreased EEG alpha activity when compared with dim light [68].

### Does nature-adapted office lighting positively affect cognitive performance?

The present study tested the hypotheses whether nature-adapted lighting conditions influences cognitive performance. For this reason, a broad spectrum of cognitive tasks was implemented in the study design. We found no effects of lighting conditions on response latencies and error rates of these tasks. Thus, the present results did not provide evidence that nature-adapted office lighting during daytime improves cognitive performance in healthy young study volunteers, as compared to standard office lighting. The differences in spectral characteristics (i.e. mEDI) may have been too small to observe light effects on cognitive performance in young healthy participants, since participants were not exposed to “bad” office lighting (200 melanopic EDI lx in the SL condition was already relatively high). However, this makes it challenging to increase cognitive performance further in healthy participants, mainly because the circadian pacemaker fully promotes wakefulness during the daytime [69].

### Does nature-adapted lighting positively influence creativity, mood and well-being?

In the study of Stefani et al. (2012), participants indicated a preference for light with dynamic clouds over light with stationary clouds for performing creative work and preferred the stationary clouds for tasks that required concentration [20]. The better ability to concentrate during static light conditions in our study provide some support for these findings. After the very first session of cognitive tasks in the morning, participants rated pleasantness in DC as lower than in the SL condition. Possibly the participants needed to adapt to the uncommon dynamic lighting fluctuations.

Interestingly, participants rated their ability to focus during the cognitive task better when they were exposed to DC. Furthermore, tension was lower in DC after completing the cognitive tasks. This could indicate that DC might reduce the mental effort for completing cognitive tasks. We hypothesized that the dynamic light scenario would be associated with a less pronounced decrease in cortisol levels in the descending phase of the circadian cortisol rhythm compared to the two static light scenarios. Overall, cortisol levels under all three light scenarios showed very similar time courses. We did not find significant differences between the three lighting conditions at any of the measured time points. On the one hand, the comparable cortisol levels might be explained by the light conditions themselves: The studies in which a light-induced effect on cortisol was observed mostly compared bright light sources (i.e., light sources with high illuminance) with a dim light control condition [26, 28]. In contrast, all three lighting scenarios in the present study had comparable illuminance levels.

We hypothesised that lighting that mimics nature in terms of colour temperature and cloud dynamics, similar to daylight, would have a direct effect on mood and circadian rhythms, which in turn would have a positive effect on cognitive performance. Therefore, our approach is biological rather than psychological. In contrast, Kaplan (1995) [70] argued in his Attention Restoration Theory (ART) that time spent in nature or viewing natural scenes helps to restore depleted attentional resources, an idea dates back to Olmsted (1865) [71]. Such restoration mitigates the negative effects of fatigue caused by directed attention on numerous other cognitive domains, such as perception, "thinking," and "acting," as well as "feeling." Our biological and Kaplan’s psychological frameworks make similar predictions, but assume somewhat different underlying mechanisms for the beneficial effects of nature/natural light. Similar to Kaplan (1995) [70], we would argue that any prolonged mental activity (such as office work) leads to fatigue, which affects productivity and well-being. The question that remains to be answered is whether naturalistic lighting can reduce the negative effects of prolonged mental activity. This would require naturalistic experimental settings where such fatigue is a notable problem, such as university classrooms, call centres or workshops.

### Is contrast sensitivity reduced under dynamic lighting?

Contrast sensitivity stayed constant during the day under DC. We observed a slight increase in contrast sensitivity under SL in the afternoon. However, the assessed contrast sensitivity using the MARS charts did not show significant differences between the light conditions in the morning and midday, which does not necessarily mean that contrast sensitivity was similar. The contrast gradations of the charts might have needed to be finer. We nevertheless used charts because we expected a greater influence of dynamic lighting when there is a direct reflection on the surface than using a self-illuminated display.

## Limitations

We are aware that this study may have several limitations. First, we only included young healthy male participants and excluded female and older participants to avoid potential effects of the menstrual cycle and age on our measurements [72]. Therefore, our results cannot be generalized for women and other age groups. Second, the time between the first (morning) and second (midday) test session was with about 5 hours quite long. Third, we did not test cognitive performance at baseline. Perhaps we were already in “saturation” with all light conditions and could not demonstrate any significant cognitive effects. Furthermore, we did not run a test session prior to light exposure, thus we cannot correct for daily fluctuations. The study design accounted for possible sequence effects among the light conditions by randomizing the order of testing days for each subject. However, the multiple assessment of that many measures did not allow for variation in task succession within a testing day without requiring a drastic increase in sample size. So we did not account for possible sequence effects that might have biased the results, for instance carry-over or interference effects, or effects of practice, adaptation, or fatigue. Although the sample size was a priori calculated to enable the discovery of small effect sizes in a within-subject design, there may be underlying issues with the reliability of the measures we applied [73] that impeded the detection of light effects on cognition. The scope of the presented piloting study is exploratory; studies following-up on our results may employ a reduced set of cognitive measures and eventually resort to performance metrics that have shown more robust to aforementioned distorting effects, maintaining reliability when applied repeatedly in close succession.

## Conclusion

Our study results showed no impact of dynamic nature-adapted lighting on subjective and objective sleepiness or cognitive performance, compared to standard workplace lighting. However, dynamic lighting was associated with a lower subjective perceived levels of tension after the cognitive tasks and less effort to concentrate than static lighting. The effects of dynamic lighting on cognitive performance, circadian physiology and well-being might take time to manifest. Thus, the effects of dynamic lighting on cognitive performance, circadian physiology, and well-being should be investigated in a more chronic situation under real-office conditions, including women and older age groups.

## Supporting information

S1 File (DOCX)
