# Supplementary material for: Effects of nature-adapted lighting solutions (“Virtual Sky”) on subjective and objective correlates of sleepiness, well-being, visual and cognitive performance at the workplace
Source: PLoS One. 2023 Aug 3;18(8):e0288690. doi: 10.1371/journal.pone.0288690 (PMC10399894; doi:10.1371/journal.pone.0288690)
Supplement: S1 File — (DOCX) [file pone.0288690.s001.docx]

**Effects of nature-adapted lighting solutions (“Virtual Sky”) on subjective and objective correlates of sleepiness, well-being, visual and cognitive performance at the workplace**

**Supplementary Material**

1. **Light Conditions**

Table S.1 Overview of the irradiance-derived light characteristics for the three

experimental light conditions. Values were calculated using the luox app [1].

| Condition | SL | SC | DC |
| --- | --- | --- | --- |
| Illuminance (lx) | 296.15 | 306.86 | 303.25 |
| CIE 1931 xy chromaticity [x] | 0.37 | 0.30 | 0.30 |
| CIE 1931 xy chromaticity [y] | 0.34 | 0.30 | 0.30 |
| CIE 1964 x₁₀y₁₀ chromaticity [x₁₀] | 0.37 | 0.31 | 0.31 |
| CIE 1964 x₁₀y₁₀ chromaticity [y₁₀] | 0.34 | 0.30 | 0.30 |
| S-cone-opic irradiance (mW ⋅ m⁻²) | 187.91 | 310.43 | 300.87 |
| M-cone-opic irradiance (mW ⋅ m⁻²) | 372.19 | 438.58 | 430.64 |
| L-cone-opic irradiance (mW ⋅ m⁻²) | 482.47 | 498.83 | 493.46 |
| Rhodopic irradiance (mW ⋅ m⁻²) | 287.63 | 423.33 | 413.01 |
| Melanopic irradiance (mW ⋅ m⁻²) | 241.33 | 398.83 | 388.23 |
| S-cone-opic EDI (lx) | 229.92 | 379.83 | 368.13 |
| M-cone-opic EDI (lx) | 255.66 | 301.26 | 295.81 |
| L-cone-opic EDI (lx) | 296.19 | 306.24 | 302.94 |
| Rhodopic EDI (lx) | 198.41 | 292.01 | 284.90 |
| Melanopic EDI (lx) | 181.97 | 300.73 | 292.74 |
| CCT (K) - Ohno, 2013 | 4056.75 | 7743.15 | 7420.95 |
| CCT (K) - Robertson, 1968 | 4056.76 | 7742.15 | 7419.81 |
| Colour Rendering Index [Ra] | 85.50 | 80.63 | 81.88 |
| TM30 - Colour Fidelity Index [Rf] | 78.56 | 74.87 | 75.89 |

# 2. All Outcome Variables

**Table S2.** Descriptive sample statistics. Repeated measures were median aggregated within each subject.

| **Test/Instrument  Outcome (Unit/Range)** | ***N*** | ***Mean*** | ***SD*** | ***Median*** | ***Skew*** | ***Transform*** |
| --- | --- | --- | --- | --- | --- | --- |
| **Karolinska Drowsiness Test (KDT)** |  |  |  |  |  |  |
| Alpha Band Power F (µV²) | 18 | 7.61 | 8.07 | 4.62 | 1.73 | log_e_(x) |
| Alpha Band Power C (µV²) | 18 | 9.71 | 9.52 | 5.89 | 1.30 | log_e_(x) |
| Alpha Band Power P (µV²) | 18 | 13.89 | 14.49 | 7.81 | 1.18 | log_e_(x) |
| Alpha Band Power O (µV²) | 18 | 7.08 | 7.50 | 5.09 | 1.75 | log_e_(x) |
| Combined Alpha Band Power (µV²) | 18 | 28.98 | 16.47 | 23.94 | 1.32 | 1/√x |
| **Psychomotor Vigilance Test (PVT)** |  |  |  |  |  |  |
| Response Latency (ms) | 18 | 283.17 | 24.28 | 280 | 0.26 | 1/x |
| **Go-NoGo** |  |  |  |  |  |  |
| Omission Errors (%) | 18 | 4.07 | 3.26 | 3.59 | 0.81 | log_e_(x) |
| Commission Errors (%) | 18 | 37.14 | 14.56 | 38.24 | -0.55 |  |
| **Word Pair Learning Test (WPLT)** |  |  |  |  |  |  |
| Mislabeled new Word Pairs (%) | 18 | 16.53 | 12.66 | 10 | 0.83 | log_e_(x) |
| Mislabeled old Word Pairs (%) | 18 | 28.89 | 13.56 | 30 | 0.74 | √x |
| Recognized new Word Pairs (%) | 18 | 66.94 | 13.30 | 65 | 0.15 |  |
| Recognized old Word Pairs (%) | 18 | 55.83 | 14.88 | 60 | -0.42 |  |
| Uncertain Response to new Word Pairs (%) | 17 | 15.74 | 7.44 | 15 | 0.58 | log_e_(x) |
| Uncertain Response to old Word Pairs (%) | 17 | 14.56 | 8.58 | 12.50 | 0.69 | log_e_(x) |
| Discrimination Index *d’* (z) | 18 | 1.24 | 0.66 | 1.15 | 0.70 |  |
| **n-Back** |  |  |  |  |  |  |
| 0-Back Error Rate (%) | 18 | 2.16 | 1.06 | 2.20 | 0.57 | √x |
| 2-Back Error Rate (%) | 18 | 4.33 | 2.10 | 4 | 1.50 | √x |
| 3-Back Error Rate (%) | 18 | 6.98 | 2.44 | 6.70 | 0.38 |  |
| **Trust Game** |  |  |  |  |  |  |
| Stake (CHF) | 18 | 6.25 | 2.64 | 6 | -0.09 |  |
| **Test for Creative Thinking – Drawing Production (TCT-DP)** | | | |  |  |  |
| Score (0 – 72) | 18 | 30.28 | 7.27 | 31 | 0.004 |  |
| **Karolinska Sleepiness Scale (KSS)** |  |  |  |  |  |  |
| Reported Sleepiness (1 – 9) | 18 | 5.33 | 1.71 | 5.50 | -0.23 |  |
| **Visual Comfort Scale (VCS)** |  |  |  |  |  |  |
| Pleasantness of Condition (-2 – 2) | 18 | 0.06 | 0.94 | 0 | -0.51 |  |
| Pleasantness of Brightness (-2 – 2) | 18 | 0.11 | 0.47 | 0 | 0.38 | √x |
| Pleasantness of Color (-2 – 2) | 18 | 0.06 | 1.06 | 0 | -0.67 |  |
| Perceived Glare (1 – 5) | 18 | 1.94 | 0.94 | 2 | 0.10 | 1/√x |
| Influence on Alertness (-2 – 2) | 18 | 0.44 | 0.70 | 1 | -0.77 |  |
| Influence on Focus (-2 – 2) | 18 | 0.22 | 0.81 | 0 | -1.01 | x² |
| **Mental Effort Rating Scale (RSME)** |  |  |  |  |  |  |
| Perceived Given Effort (%) | 18 | 69.37 | 15.10 | 71.51 | -0.44 | x² |
| Satisfaction With Performance (%) | 18 | 44.23 | 17.79 | 42.35 | 0.13 | √x |
| Focussing Effort (%) | 18 | 73.69 | 13.28 | 74.59 | 0.04 | x² |
| Exhaustion (%) | 18 | 64.55 | 13.01 | 66.40 | -0.20 |  |
| Motivation (%) | 18 | 57.89 | 23.45 | 55.99 | 0.24 |  |
| Effort Relative to Previous Testings (%) | 18 | 64.21 | 17.70 | 61.52 | 0.08 |  |
|  |  |  |  |  |  |  |
|  |  |  |  |  |  |  |
|  |  |  |  |  |  |  |
| **Subjective Mood and Well-Being** |  |  |  |  |  |  |
| Momentary Tension (%) | 18 | 37.78 | 13.16 | 34.16 | 0.79 | √x |
| Physical Comfort (%) | 18 | 64.42 | 13.57 | 66.78 | -0.34 | x² |
| Momentary Fatigue (%) | 18 | 53.63 | 11.09 | 53.22 | -0.29 |  |
| Momentary Sleepiness (%) | 18 | 61.04 | 8.56 | 58.81 | 0.77 |  |
| Current Mood (%) | 18 | 62.10 | 12.79 | 58.73 | 0.71 |  |
| **Mars Letter Contrast Sensitivity Test (MARS)** | |  |  |  |  |  |
| Sensitivity to Contrast (0.04 – 1.92) | 18 | 1.81 | 0.08 | 1.84 | -1.10 | 1/√(2 - x) |
| **Pittsburgh Sleep Questionnaire** |  |  |  |  |  |  |
| Score (0 – 5) | 18 | 3.17 | 1.20 | 3 | -0.69 | x² |
| **Munich Chronotype questionnaire (MCTQ)** | | | |  |  |  |
| Midsleep on Free Days, adjusted (hrs) | 18 | 4.38 | 0.97 | 4.37 | 0.07 |  |

*Note.* SD: standard deviation; log_e_: natural logarithm; √: square root

# 3. Statistical Analysis

Statistical analyses were performed using the R statistical computing environment (Version 4.0.3, R Core Team, 2020). The packages *lmerTest* (v3.1-3, Kuznetsova et al., 2017) and *lme4* (v1.1-27.1, Bates et al., 2015) were employed for the linear mixed models, as well as the non-linear mixed modelling of the salivary cortisol data. For model diagnostics and performance evaluation the package *performance* (v0.7.0, Lüdecke et al., 2020) was used.

LMM fixed effects were tested for significance by one- or two-factorial analysis of deviance (AoD) Wald-type chi squared (Χ²) statistics provided by the *Anova* function of the *car* package (v3.0-12, Fox & Weisberg, 2019).

Cohen’s omega squared (ω^2^) as calculated by the *effectsize* package (v0.4.5, Ben-Shachar et al., 2020) is provided as effect size measure for the LMM fixed effects; it can be interpreted as follows: very small effect: ω^2^ < .01 small effect: ω^2^ ≥ .01, medium effect: ω^2^ ≥ .06, large effect: ω^2^ ≥ .14 (analogous to eta squared, Cohen, 1988); for models with more than one fixed effect, partial omega squared (ω_p_^2^) is reported, the same interpretation applies. Contrast testing of significant AoD fixed effects was performed using the *emmeans* package (v1.5.4, Lenth, 2021) with Šidák and Tukey adjustment for multiple testing; only simple main effects were of interest.

In case of unmet LMM assumptions of homoscedasticity and homogeneity of variances the respective dependent variable was submitted to a Box-Cox analysis [10] and a common power transformation was applied to the DV depending on lambda value (λ). Extremely left skewed distributions were mirrored prior to transformation by subtracting all values from the first whole number larger than the maximum value.

If unresolvable heteroscedasticity, heterogeneous group variances, or autocorrelation were still present in the selected model, AoD and contrast analysis were performed using a cluster-robust variance-covariance matrix (CR2 adjustment for small samples) as provided by the R-package *clubSandwich* (v 0.5.3, Pustejovsky & Tipton, 2018).

# 4. Results

## Supplementary Figures

### Cognitive Performance

**A)**

**B)**

**C)**


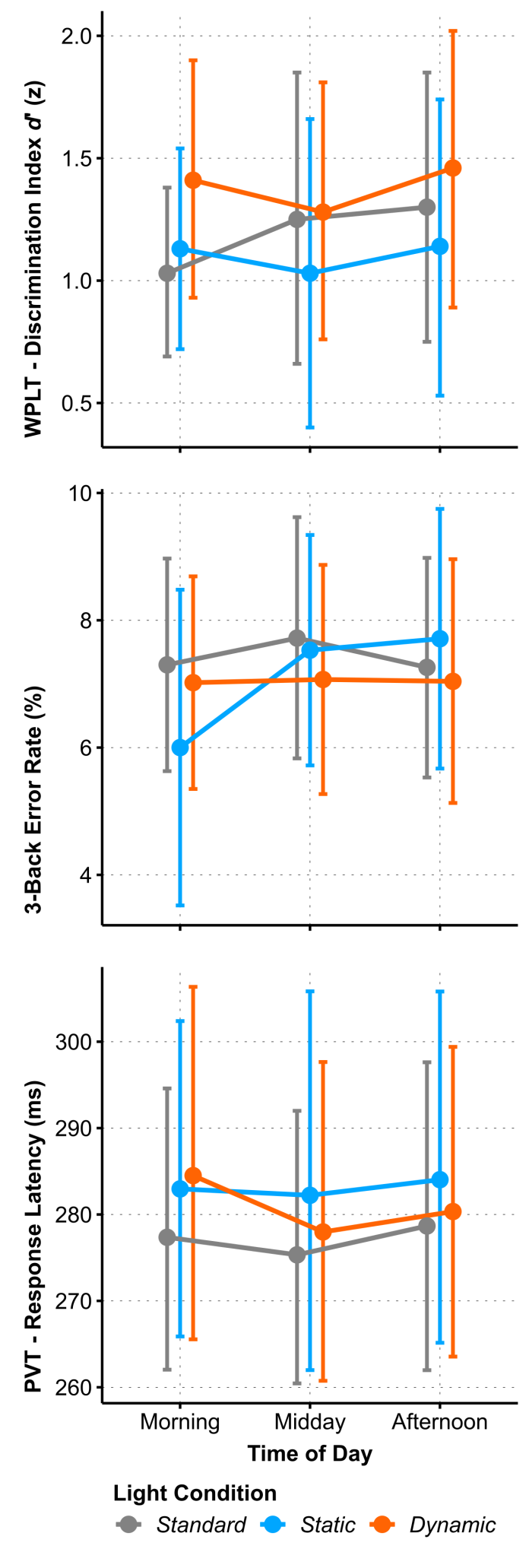
**Figure S1.** Interaction plots of estimated means for A) WPLT Discrimination Index *d*’ (z), B) 3-Back *Error Rate* (%), and C) *Response Latency* in the Psychomotor Vigilance Task (PVT, ms) between *Light Conditions* across *Time of Day*. Error bars indicate adjusted 95 % confidence intervals.

### Subjective Sleepiness – Karolinska Sleepiness Scale (KSS)


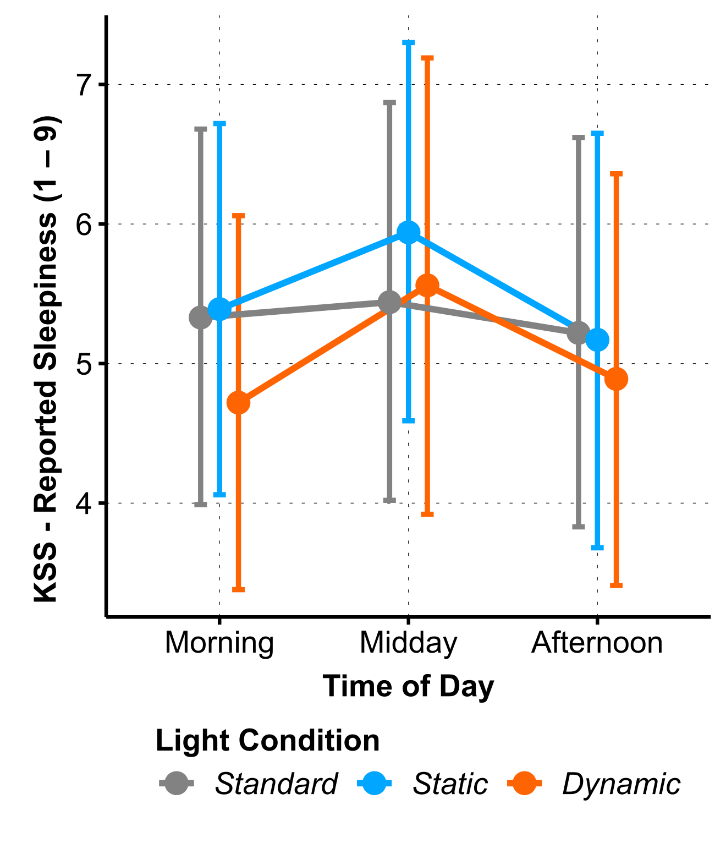


**Figure S2.** Interaction plots of estimated means of Subjective Sleepiness Ratings (KSS 1-9) between *Light Conditions* across *Time of Day*. Error bars indicate adjusted 95 % confidence intervals.

### Karolinska Drowsiness Test (KDT) – FFT Profiles


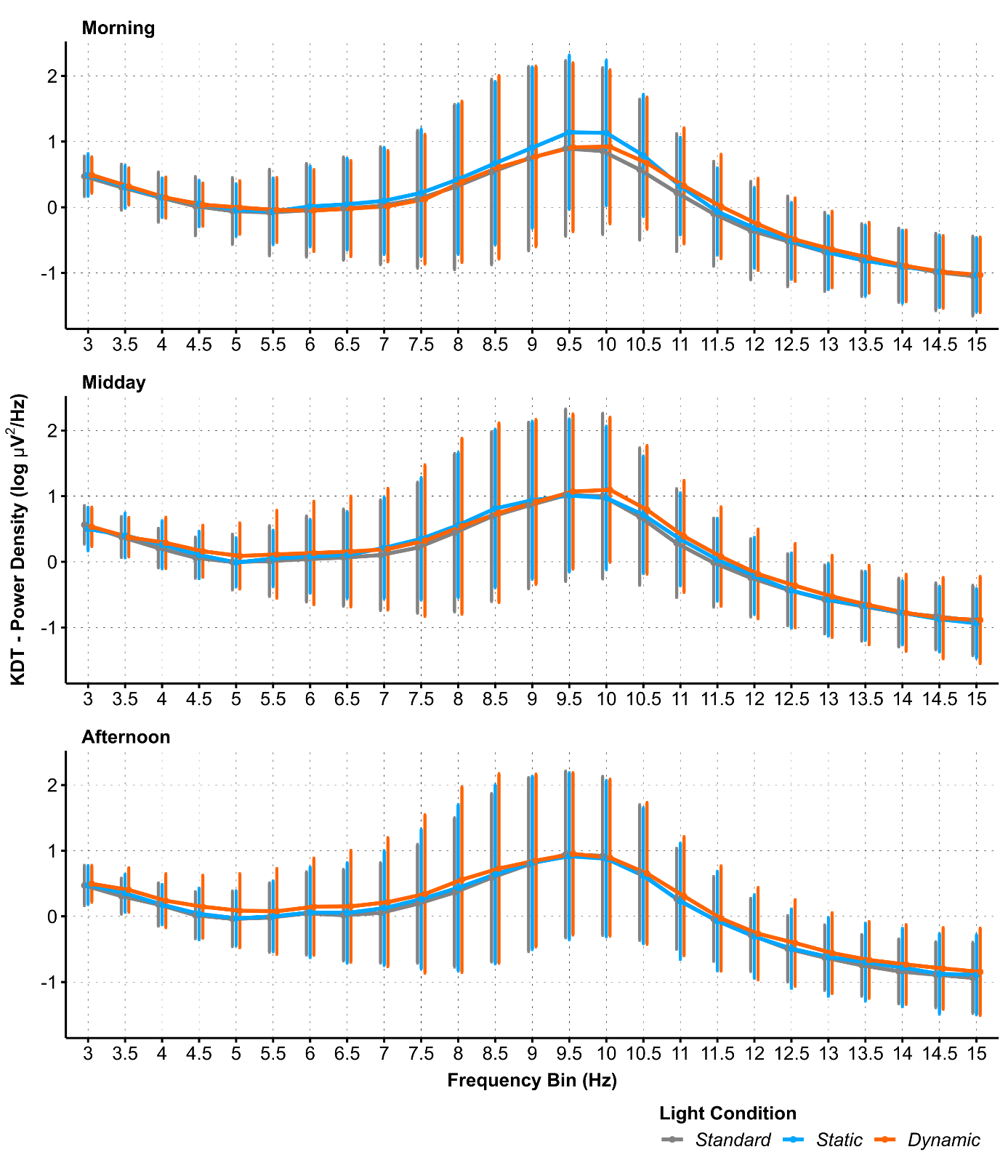


**Figure S3.** KDT EEG power density profiles (3 – 15 Hz) at the combined parietal electrodes (P3, Pz, P4) for the three Light Conditions (lines) measured at three Times of Day (panels). Power density scale is log-transformed; vertical bars indicate ± standard deviation.

## Analysis of Deviance Model Parameters

This section assembles tables with the analysis of deviance (AoD) parameters of all linear mixed models. For outcome variables with significant effects, the selected model as reported in the main article is displayed. All AoDs are represented by the fully crossed model.

### KDT

**Table S3.** AoD model parameters of the KDT Alpha Band Power Band for regions F, C, P, and O .

| **Outcome (Unit) Effect** | **Χ²** | ***Df*** | ***p*** | **ω_p_²** | **ω_p_² Interpret.** | ***R²* cond.** | ***R²* marg.** | ***ICC*** |
| --- | --- | --- | --- | --- | --- | --- | --- | --- |
| **Alpha Band Power F (µV²)** |  |  |  |  |  |  |  |  |
| (Intercept) | 58.71 | 1 | <.001 |  |  | 0.88 | 0.01 | 088 |
| Light Condition | 2.07 | 2 | .36 | 0.01 | small |  |  |  |
| Time of Day | 2.70 | 2 | .26 | -0.002 | very small |  |  |  |
| Light Condition × Time of Day | 2.14 | 4 | .71 | -0.02 | very small |  |  |  |
| **Alpha Band Power C (µV²)** |  |  |  |  |  |  |  |  |
| (Intercept) | 71.99 | 1 | <.001 |  |  | 0.93 | <0.01 | 0.93 |
| Light Condition | 2.27 | 2 | .32 | 0.02 | small |  |  |  |
| **Selected Model:**  Time of Day | 9.14 | 2 | **.01** | 0.02 | small |  |  |  |
| (Intercept) | 71.99 | 1 | <.001 |  |  | 0.93 | 0.01 | 0.93 |
| Light Condition | 2.27 | 2 | .32 | 0.02 | small |  |  |  |
| Time of Day | 9.14 | 2 | .01 | 0.02 | small |  |  |  |
| Light Condition × Time of Day | 7.29 | 4 | .12 | -0.01 | very small |  |  |  |
| **Alpha Band Power P (µV²)** |  |  |  |  |  |  |  |  |
| (Intercept) | 68.91 | 1 | <.001 |  |  | 0.95 | <0.01 | 0.95 |
| Light Condition | 1.13 | 2 | .57 | 0.006 | very small |  |  |  |
| Time of Day | 6.70 | 2 | .04 | 0.02 | small |  |  |  |
| Light Condition × Time of Day | 9.69 | 4 | **.046** | -0.01 | very small |  |  |  |
| **Alpha Band Power O (µV²)** |  |  |  |  |  |  |  |  |
| (Intercept) | 50.48 | 1 | <.001 |  |  | 0.91 | <0.01 | 0.91 |
| **Selected Model:**  Time of Day | 7.09 | 2 | **.03** | 0.01 | very small |  |  |  |
| (Intercept) | 50.48 | 1 | <.001 |  |  | 0.91 | 0.00 | 0.91 |
| Light Condition | 0.72 | 2 | .70 | 0.00 | very small |  |  |  |
| Time of Day | 7.09 | 2 | .029 | 0.01 | very small |  |  |  |
| Light Condition × Time of Day | 0.84 | 4 | .93 | -0.02 | very small |  |  |  |

Note. *Df*: degrees of freedom of Χ²; *ICC*: intra class correlation, variance proportion of random effects

### Subjective Sleepiness (KSS), Momentary Sleepiness (VAS), Mood and Well-Being

**Table S4.** AoD model parameters of Subjective Sleepiness (KSS), Momentary Sleepiness (VAS), and Subjective Mood and Well-Being.

| **Outcome (Unit) Effect** | **Χ²** | ***Df*** | ***p*** | **ω_p_²** | **ω_p_² Interpret.** | ***R²* cond.** | ***R²* marg.** | ***ICC*** |
| --- | --- | --- | --- | --- | --- | --- | --- | --- |
| **KSS** |  |  |  |  |  |  |  |  |
| (Intercept) | 248.13 | 1 | <.001 |  |  | 0.40 | 0.03 | 0.39 |
| Light Condition | 1.37 | 2 | .50 | < 0.01 | very small |  |  |  |
| Time of Day | 3.27 | 2 | .19 | 0.01 | small |  |  |  |
| Light Condition × Time of Day | 1.67 | 4 | .80 | -0.02 | very small |  |  |  |
| **Momentary Sleepiness (%)** |  |  |  |  |  |  |  |  |
| (Intercept) | 687.30 | 1 | <.001 |  |  | 0.21 | 0.02 | 0.20 |
| **Selected Model:**  Time of Day | 6.15 | 2 | **.046** | 0.01 | small |  |  |  |
| (Intercept) | 687.30 | 1 | <.001 |  |  | 0.22 | 0.03 | 0.19 |
| Light Condition | 1.22 | 2 | .54 | 0.00 | very small |  |  |  |
| Time of Day | 6.15 | 2 | .046 | 0.01 | small |  |  |  |
| Light Condition × Time of Day | 1.48 | 4 | .83 | -0.02 | very small |  |  |  |
| **Momentary Tension (%)** |  |  |  |  |  |  |  |  |
| (Intercept) | 1111.31 | 1 | <.001 |  |  | 0.24 | 0.06 | 0.19 |
| Light Condition | 9.56 | 2 | **.008** | 0.05 | small |  |  |  |
| Time of Day | 1.73 | 2 | .42 | < 0.01 | very small |  |  |  |
| Light Condition × Time of Day | 1.43 | 4 | .84 | -0.02 | very small |  |  |  |
| **Physical Comfort (%)** |  |  |  |  |  |  |  |  |
| (Intercept) | 161.64 | 1 | <.001 |  |  | 0.42 | 0.02 | 0.41 |
| Light Condition | 1.47 | 2 | .48 | < 0.01 | very small |  |  |  |
| Time of Day | 0.02 | 2 | .99 | -0.01 | very small |  |  |  |
| Light Condition × Time of Day | 5.71 | 4 | .22 | -0.01 | very small |  |  |  |
| **Momentary Fatigue (%)** |  |  |  |  |  |  |  |  |
| (Intercept) | 705.77 | 1 | <.001 |  |  | 0.25 | 0.06 | 0.20 |
| **Selected Model:**  Time of Day | 13.41 | 2 | **.001** | 0.07 | medium |  |  |  |
| (Intercept) | 705.77 | 1 | <.001 |  |  | 0.26 | 0.08 | 0.20 |
| Light Condition | 0.20 | 2 | .91 | -0.01 | very small |  |  |  |
| Time of Day | 13.41 | 2 | .001 | 0.08 | medium |  |  |  |
| Light Condition × Time of Day | 5.71 | 4 | .22 | 0.00 | very small |  |  |  |
| **Current Mood (%)** |  |  |  |  |  |  |  |  |
| (Intercept) | 509.31 | 1 | <.001 |  |  | 0.51 | 0.03 | 0.50 |
| **Selected Model:**  Time of Day | 17.93 | 2 | **<.001** | 0.04 | small |  |  |  |
| (Intercept) | 509.31 | 1 | <.001 |  |  | 0.51 | 0.03 | 0.49 |
| Light Condition | 0.39 | 2 | .82 | -0.01 | very small |  |  |  |
| Time of Day | 17.93 | 2 | <.001 | 0.04 | small |  |  |  |
| Light Condition × Time of Day | 6.64 | 4 | .16 | -0.01 | very small |  |  |  |

Note. *Df*: degrees of freedom of Χ²; *ICC*: intra class correlation, variance proportion of random effects

### Cognitive Performance

**Table S5.** AoD model parameters of the cognitive performance variables.

| **Outcome (Unit) Effect** | **Χ²** | ***Df*** | ***p*** | **ω_p_²** | **ω_p_² Interpret.** | ***R²* cond.** | ***R²* marg.** | ***ICC*** |
| --- | --- | --- | --- | --- | --- | --- | --- | --- |
| **n-Back - 0-Back Error Rate (%)** |  |  |  |  |  |  |  |  |
| (Intercept) | 353.97 | 1 | <.001 |  |  | 0.53 | 0.02 | 0.52 |
| Light Condition | 2.03 | 2 | .36 | < 0.01 | very small |  |  |  |
| Time of Day | 5.03 | 2 | .08 | 0.01 | small |  |  |  |
| Light Condition × Time of Day | 4.14 | 4 | .39 | -0.01 | very small |  |  |  |
| **n-Back - 2-Back Error Rate (%)** |  |  |  |  |  |  |  |  |
| (Intercept) | 403.00 | 1 | <.001 |  |  | 0.52 | 0.02 | 0.51 |
| Light Condition | 0.67 | 2 | .72 | < 0.01 | very small |  |  |  |
| Time of Day | 0.62 | 2 | .73 | -0.01 | very small |  |  |  |
| Light Condition × Time of Day | 4.53 | 4 | .34 | 0.01 | very small |  |  |  |
| **n-Back - 3-Back Error Rate (%)** |  |  |  |  |  |  |  |  |
| (Intercept) | 201.95 | 1 | <.001 |  |  | 0.56 | 0.03 | 0.55 |
| Light Condition | 0.67 | 2 | .72 | < 0.01 | very small |  |  |  |
| Time of Day | 4.71 | 2 | .09 | 0.01 | small |  |  |  |
| Light Condition × Time of Day | 7.57 | 4 | .11 | 0.01 | small |  |  |  |
| **PVT - Response Latency (ms)** |  |  |  |  |  |  |  |  |
| (Intercept) | 2715.52 | 1 | <.001 |  |  | 0.72 | 0.01 | 0.72 |
| Light Condition | 2.68 | 2 | .26 | 0.02 | small |  |  |  |
| Time of Day | 1.88 | 2 | .39 | < 0.01 | very small |  |  |  |
| Light Condition × Time of Day | 2.22 | 4 | .69 | -0.02 | very small |  |  |  |
| **Go-NoGo - Omission Errors (%)** |  |  |  |  |  |  |  |  |
| (Intercept) | 113.19 | 1 | <.001 |  |  | 0.63 | 0.02 | 0.62 |
| Light Condition | 2.64 | 2 | .27 | 0.01 | very small |  |  |  |
| Time of Day | 5.14 | 2 | .08 | 0.03 | small |  |  |  |
| Light Condition × Time of Day | 2.41 | 4 | .66 | -0.02 | very small |  |  |  |
| **Go-NoGo - Comission Errors (%)** |  |  |  |  |  |  |  |  |
| (Intercept) | 117.39 | 1 | <.001 |  |  | 0.71 | 0.01 | 0.70 |
| **Selected Model:**  Time of Day | 6.21 | 2 | **.045** | 0.04 | small |  |  |  |
| (Intercept) | 117.02 | 1 | <.001 |  |  | 0.70 | 0.02 | 0.70 |
| Light Condition | 2.02 | 2 | .36 | 0.00 | very small |  |  |  |
| Time of Day | 6.17 | 2 | .046 | 0.04 | small |  |  |  |
| Light Condition × Time of Day | 0.41 | 4 | .98 | -0.03 | very small |  |  |  |
| **WPLT - Mislabelled new Word Pairs (%)** |  |  |  |  |  |  |  |  |
| (Intercept) | 284.23 | 1 | <.001 |  |  | 0.61 | 0.02 | 0.60 |
| Light Condition | 3.54 | 2 | .17 | 0.02 | small |  |  |  |
| Time of Day | 2.90 | 2 | .24 | 0.01 | small |  |  |  |
| Light Condition × Time of Day | 1.14 | 4 | .89 | -0.03 | very small |  |  |  |
| **WPLT - Mislabelled old Word Pairs (%)** |  |  |  |  |  |  |  |  |
| (Intercept) | 362.48 | 1 | <.001 |  |  | 0.51 | 0.02 | 0.50 |
| Light Condition | 0.20 | 2 | .90 | -0.01 | very small |  |  |  |
| Time of Day | 2.86 | 2 | .24 | < 0.01 | very small |  |  |  |
| Light Condition × Time of Day | 3.48 | 4 | .48 | < 0.01 | very small |  |  |  |
|  |  |  |  |  |  |  |  |  |
| **WPLT - Recognized new Word Pairs (%)** |  |  |  |  |  |  |  |  |
| (Intercept) | 411.91 | 1 | <.001 |  |  | 0.58 | 0.02 | 0.57 |
| Light Condition | 2.37 | 2 | .31 | 0.01 | small |  |  |  |
| Time of Day | 4.30 | 2 | .12 | 0.01 | small |  |  |  |
| Light Condition × Time of Day | 4.12 | 4 | .39 | -0.02 | very small |  |  |  |
| **WPLT - Recognized old Word Pairs (%)** |  |  |  |  |  |  |  |  |
| (Intercept) | 261.96 | 1 | <.001 |  |  | 0.53 | 0.03 | 0.52 |
| Light Condition | 2.68 | 2 | .26 | 0.01 | very small |  |  |  |
| Time of Day | 0.38 | 2 | .83 | -0.01 | very small |  |  |  |
| Light Condition × Time of Day | 9.11 | 4 | .06 | 0.01 | very small |  |  |  |
| **WPLT - Discrimination Index *d*' (sd)** |  |  |  |  |  |  |  |  |
| (Intercept) | 76.53 | 1 | <.001 |  |  | 0.56 | 0.03 | 0.54 |
| Light Condition | 5.36 | 2 | .07 | 0.04 | small |  |  |  |
| Time of Day | 1.66 | 2 | .44 | < 0.01 | very small |  |  |  |
| Light Condition × Time of Day | 7.43 | 4 | .11 | -0.01 | very small |  |  |  |

Note. *Df*: degrees of freedom of Χ²; *ICC*: intra class correlation, variance proportion of random effects

### Test for Creative Thinking – Drawing Production (TCT-DP) & Trust Game

**Table S6.** AoD model parameters of the Test for Creative Thinking – Drawing Production (TCT-DP) & Trust Game. These Tests were performed only once per light condition.

| **Outcome (Unit) Effect** | **Χ²** | ***Df*** | ***p*** | **ω_p_²** | **ω_p_² Interpret.** | ***R²* cond.** | ***R²* marg.** | ***ICC*** |
| --- | --- | --- | --- | --- | --- | --- | --- | --- |
| **Test for Creative Thinking (0 – 72)** |  |  |  |  |  |  |  |  |
| (Intercept) | 330.32 | 1 | <.001 |  |  | 0.34 | 0.004 | 0.33 |
| Light Condition | 0.31 | 2 | .86 | -0.05 | very small |  |  |  |
| **Trustgame - Stake (CHF)** |  |  |  |  |  |  |  |  |
| (Intercept) | 114.13 | 1 | <.001 |  |  | 0.69 | 0.02 | 0.68 |
| Light Condition | 3.19 | 2 | .20 | 0.04 | very small |  |  |  |

Note. *Df*: degrees of freedom of Χ²; *ICC*: intra class correlation, variance proportion of random effects

### Subjective Effort Rating (RSME)

**Table S7.** AoD model parameters of the Subjective Effort Rating measures (RSME).

| **Outcome (Unit) Effect** | **Χ²** | ***Df*** | ***p*** | **ω_p_²** | **ω_p_² Interpret.** | ***R²* cond.** | ***R²* marg.** | ***ICC*** |
| --- | --- | --- | --- | --- | --- | --- | --- | --- |
| **Perceived Given Effort (%) (-2 – 2)** |  |  |  |  |  |  |  |  |
| (Intercept) | 130.96 | 1 | <.001 |  |  | 0.59 | 0.03 | 0.57 |
| **Selected Model:**  Time of Day | 9.08 | 2 | .01 | 0.07 | medium |  |  |  |
| (Intercept) | 130.96 | 1 | <.001 |  |  | 0.59 | 0.05 | 0.57 |
| Light Condition | 3.56 | 2 | .17 | 0.01 | small |  |  |  |
| Time of Day | 9.08 | 2 | .011 | 0.08 | medium |  |  |  |
| Light Condition × Time of Day | 4.45 | 4 | .35 | -0.01 | very small |  |  |  |
| **Satisfaction With Performance (%)** |  |  |  |  |  |  |  |  |
| (Intercept) | 615.33 | 1 | <.001 |  |  | 0.34 | 0.02 | 0.33 |
| Light Condition | 0.69 | 2 | .71 | -0.01 | very small |  |  |  |
| Time of Day | 2.50 | 2 | .29 | < 0.01 | very small |  |  |  |
| Light Condition × Time of Day | 3.37 | 4 | .50 | -0.02 | very small |  |  |  |
| **Focussing Effort (%)** |  |  |  |  |  |  |  |  |
| (Intercept) | 140.92 | 1 | <.001 |  |  | 0.67 | 0.03 | 0.66 |
| **Selected Model:**  Light Condition | 12.71 | 2 | .002 | 0.08 | medium |  |  |  |
| (Intercept) | 140.92 | 1 | <.001 |  |  | 0.67 | 0.03 | 0.66 |
| Light Condition | 12.71 | 2 | .002 | 0.08 | medium |  |  |  |
| Time of Day | 0.60 | 2 | .74 | -0.01 | very small |  |  |  |
| Light Condition × Time of Day | 6.20 | 4 | .18 | -0.01 | very small |  |  |  |
| **Exhaustion (%)** |  |  |  |  |  |  |  |  |
| (Intercept) | 627.22 | 1 | <.001 |  |  | 0.27 | 0.05 | 0.23 |
| **Selected Model:**  Time of Day | 7.18 | 2 | .03 | 0.06 | small |  |  |  |
| (Intercept) | 627.22 | 1 | <.001 |  |  | 0.28 | 0.07 | 0.22 |
| Light Condition | 0.98 | 2 | .61 | 0.00 | very small |  |  |  |
| Time of Day | 7.18 | 2 | .028 | 0.06 | medium |  |  |  |
| Light Condition × Time of Day | 4.72 | 4 | .32 | -0.01 | very small |  |  |  |
| **Motivation (%)** |  |  |  |  |  |  |  |  |
| (Intercept) | 130.00 | 1 | <.001 |  |  | 0.73 | 0.04 | 0.71 |
| **Selected Model:**  Time of Day | 31.36 | 2 | <.001 | 0.12 | medium |  |  |  |
| (Intercept) | 129.49 | 1 | <.001 |  |  | 0.72 | 0.04 | 0.71 |
| Light Condition | 0.05 | 2 | .98 | -0.01 | very small |  |  |  |
| Time of Day | 30.65 | 2 | <.001 | 0.13 | medium |  |  |  |
| Light Condition × Time of Day | 4.08 | 4 | .40 | -0.01 | very small |  |  |  |
| **Effort Relative to Prev. Testings (%)** |  |  |  |  |  |  |  |  |
| (Intercept) | 325.60 | 1 | <.001 |  |  | 0.33 | 0.01 | 0.32 |
| Light Condition | 2.71 | 2 | .26 | < 0.01 | very small |  |  |  |
| Time of Day | 0.41 | 1 | .52 | -0.01 | very small |  |  |  |
| Light Condition × Time of Day | 0.01 | 2 | 1.00 | -0.02 | very small |  |  |  |

Note. *Df*: degrees of freedom of Χ²; *ICC*: intra class correlation, variance proportion of random effects

### Visual Comfort and Contrast Sensitivity

**Table S8.** AoD model parameters of the Visual Comfort and Contrast Sensitivity measures.

| **Outcome (Unit) Effect** | **Χ²** | ***Df*** | ***p*** | **ω_p_²** | **ω_p_² Interpret.** | ***R²* cond.** | ***R²* marg.** | ***ICC*** |
| --- | --- | --- | --- | --- | --- | --- | --- | --- |
| **VCS - Pleasantness of Conditions (-2 – 2)** | |  |  |  |  |  |  |  |
| (Intercept) | 0.14 | 1 | .71 |  |  | 0.65 | 0.09 | 0.62 |
| Light Condition | 9.71 | 2 | .008 | 0.10 | medium |  |  |  |
| Time of Day | 1.37 | 2 | .50 | -0.01 | very small |  |  |  |
| Light Condition × Time of Day | 14.92 | 4 | .005 | 0.08 | medium |  |  |  |
| **VCS - Pleasantness of Brightness (-2 – 2)** |  |  |  |  |  |  |  |  |
| (Intercept) | 253.19 | 1 | <.001 |  |  | 0.87 | 0.04 | 0.86 |
| Light Condition | 3.04 | 2 | .22 | 0.12 | medium |  |  |  |
| Time of Day | 1.41 | 2 | .49 | < 0.01 | very small |  |  |  |
| Light Condition × Time of Day | 2.92 | 4 | .57 | -0.07 | very small |  |  |  |
| **VCS - Pleasantness of Color (-2 – 2)** |  |  |  |  |  |  |  |  |
| (Intercept) | 0.00 | 1 | .98 |  |  | 0.74 | 0.03 | 0.74 |
| Light Condition | 4.66 | 2 | .10 | 0.10 | medium |  |  |  |
| Time of Day | 0.47 | 2 | .79 | -0.02 | very small |  |  |  |
| Light Condition × Time of Day | 2.85 | 4 | .58 | -0.04 | very small |  |  |  |
| **VCS - Perceived Glare (1 – 5)** |  |  |  |  |  |  |  |  |
| (Intercept) | 529.05 | 1 | <.001 |  |  | 0.51 | 0.01 | 0.51 |
| Light Condition | 0.19 | 2 | .91 | -0.01 | very small |  |  |  |
| Time of Day | 0.41 | 2 | .81 | -0.01 | very small |  |  |  |
| Light Condition × Time of Day | 6.50 | 4 | .17 | -0.01 | very small |  |  |  |
| **VCS - Influence on Alertness (-2 – 2)** |  |  |  |  |  |  |  |  |
| (Intercept) | 2.55 | 1 | .11 |  |  | 0.56 | 0.06 | 0.53 |
| Light Condition | 3.78 | 2 | .15 | 0.06 | medium |  |  |  |
| Time of Day | 2.69 | 2 | .26 | -0.01 | very small |  |  |  |
| Light Condition × Time of Day | 9.66 | 4 | .047 | -0.02 | very small |  |  |  |
| **VCS - Influence on Focus (-2 – 2)** |  |  |  |  |  |  |  |  |
| (Intercept) | 82.16 | 1 | <.001 |  |  | 0.67 | 0.07 | 0.65 |
| **Selected Model:**  Light Condition | 10.53 | 2 | .005 | 0.15 | large |  |  |  |
| (Intercept) | 82.41 | 1 | <.001 |  |  | 0.66 | 0.08 | 0.63 |
| Light Condition | 11.39 | 2 | .003 | 0.16 | large |  |  |  |
| Time of Day | 3.35 | 2 | .19 | -0.01 | very small |  |  |  |
| Light Condition × Time of Day | 8.04 | 4 | .09 | -0.02 | very small |  |  |  |
| **MARS - Contrast Sensitivity (0.04 – 1.92)** |  |  |  |  |  |  |  |  |
| (Intercept) | 812.66 | 1 | <.001 |  |  | 0.50 | 0.05 | 0.48 |
| Light Condition | 1.24 | 2 | .54 | < 0.01 | very small |  |  |  |
| Time of Day | 4.06 | 2 | .13 | < 0.01 | very small |  |  |  |
| Light Condition × Time of Day | 37.08 | 4 | <.001 | 0.05 | small |  |  |  |

Note. *Df*: degrees of freedom of Χ²; *ICC*: intra class correlation, variance proportion of random effects

## False Discovery Rate

**Table S9.** On the level of the linear mixed models, a *q*-value based analysis of false discovery rate (FDR; Storey, 2002 [12]) using the *p*-values for a total of 81 tested AoD effects was conducted. FDR π0 of was 0.475. The table shows only the 11 tests that were significant with *p* < .05, sorted by ascending *p*-values; eliminated effects in bold; *p*-values and *q*-values < .05 in red.

| **Outcome** | **Effect** | **Χ²** | ***Df*** | ***p*-value** | **omega²** | **effect size** | ***q*-value** |
| --- | --- | --- | --- | --- | --- | --- | --- |
| RSME - Motivation | Time of Day | 31.36 | 2 | <.001 | 0.124 | medium | <0.0001 |
| MARS - Contrast Sensitivity | Light Condition × Time of Day | 37.08 | 4 | <.001 | 0.052 | small | <0.0001 |
| Current Mood | Time of Day | 17.93 | 2 | <.001 | 0.042 | small | 0.002 |
| Momentary Fatigue | Time of Day | 13.41 | 2 | .001 | 0.074 | medium | 0.012 |
| RSME - Focussing Effort | Light Condition | 12.71 | 2 | .002 | 0.075 | medium | 0.013 |
| VCS - Pleasantness of Conditions | Light Condition × Time of Day | 14.92 | 4 | .005 | 0.079 | medium | 0.029 |
| VCS - Influence on Focus | Light Condition | 10.53 | 2 | .005 | 0.155 | large | 0.029 |
| Momentary Tension | Light Condition | 9.76 | 2 | .008 | 0.051 | small | 0.033 |
| VCS - Pleasantness of Conditions | Light Condition | 9.71 | 2 | .008 | 0.105 | medium | 0.033 |
| KDT - Alpha Band Power C | Time of Day | 9.14 | 2 | .010 | 0.023 | small | 0.038 |
| RSME - Perceived Given Effort | Time of Day | 9.08 | 2 | .011 | 0.074 | medium | 0.038 |
| **RSME - Exhaustion** | **Time of Day** | **7.18** | **2** | **.028** | **0.059** | **small** | **0.09** |
| **KDT - Alpha Band Power O** | **Time of Day** | **7.09** | **2** | **.029** | **0.006** | **very small** | **0.09** |
| **KDT - Alpha Band Power P** | **Time of Day** | **6.70** | **2** | **.035** | **0.018** | **small** | **0.10** |
| **Go-NoGo - Commission Errors** | **Time of Day** | **6.21** | **2** | **.045** | **0.039** | **small** | **0.10** |
| **KDT - Alpha Band Power P** | **Light Condition × Time of Day** | **9.69** | **4** | **.046** | **-0.006** | **very small** | **0.10** |
| **VAS - Momentary Sleepiness** | **Time of Day** | **6.15** | **2** | **.046** | **0.014** | **small** | **0.10** |
| **VCS - Influence on Alertness** | **Light Condition × Time of Day** | **9.66** | **4** | **.047** | **-0.017** | **very small** | **0.10** |

# 5. References

1. Spitschan M, Mead J, Roos C, Lowis C, Griffiths B, Mucur P, et al. luox: validated reference open-access and open-source web platform for calculating and sharing physiologically relevant quantities for light and lighting. Wellcome Open Res. 2022;6: 69. doi:10.12688/wellcomeopenres.16595.3

2. R Core Team. R: A language and environment for statistical computing. R Foundation for Statistical Computing. Vienna, Austria; 2020.

3. Kuznetsova A, Brockhoff PB, Christensen RHB. lmerTest Package: Tests in Linear Mixed Effects Models. J Stat Softw. 2017;82. doi:10.18637/jss.v082.i13

4. Bates D, Mächler M, Bolker B, Walker S. Fitting Linear Mixed-Effects Models Using lme4. J Stat Softw. 2015;67: 1–48. doi:10.18637/jss.v067.i01

5. Lüdecke D, Makowski D, Waggoner P, Patil I. performance: Assessment of Regression Models Performance. CRAN. 2020. doi:10.5281/zenodo.3952174

6. Fox J, Weisberg S. An R Companion to Applied Regression. Third edition. Los Angeles: SAGE; 2019.

7. Ben-Shachar MS, Lüdecke D, Makowski D. effectsize: Estimation of Effect Size Indices and Standardized Parameters. J Open Source Softw. 2020;5: 2815. doi:10.21105/joss.02815

8. Cohen J. Statistical power analysis for the behavioral sciences. 2nd ed. Hillsdale, N.J: L. Erlbaum Associates; 1988.

9. Lenth RV. emmeans: Estimated Marginal Means, aka Least-Squares Means. 2021. Available: https://CRAN.R-project.org/package=emmeans

10. Box GEP, Cox DR. An Analysis of Transformations. J R Stat Soc Ser B Methodol. 1964;26: 211–252.

11. Pustejovsky JE, Tipton E. Small-Sample Methods for Cluster-Robust Variance Estimation and Hypothesis Testing in Fixed Effects Models. J Bus Econ Stat. 2018;36: 672–683. doi:10.1080/07350015.2016.1247004

12. Storey JD. A Direct Approach to False Discovery Rates. J R Stat Soc Ser B Stat Methodol. 2002;64: 479–498. doi:10.1111/1467-9868.00346
